# Sense of agency at a gaze-contingent display with jittery temporal delay

**DOI:** 10.3389/fpsyg.2024.1364076

**Published:** 2024-05-17

**Authors:** Junhui Kim, Takako Yoshida

**Affiliations:** Department of Mechanical Engineering, School of Engineering, Tokyo Institute of Technology, Tokyo, Japan

**Keywords:** eye movements, perception and action, sense of agency, HCI—human-computer interaction, action refinement, oculomotor control

## Abstract

**Introduction:**

Inconsistent jittery temporal delays between action and subsequent feedback, prevalent in network-based human–computer interaction (HCI), have been insufficiently explored, particularly regarding their impact on the sense of agency (SoA). This study investigates the SoA in the context of eye-gaze HCI under jittery delay conditions.

**Methods:**

Participants performed a visual search for Chinese characters using a biresolutional gaze-contingent display, which displayed a high-resolution image in the central vision and a low-resolution in the periphery. We manipulated the delay between eye movements and display updates using a truncated normal distribution (μ to μ + 2 σ) with μ ranging from 0 to 400 ms and σ fixed at 50 ms. Playback of recorded gaze data provided a non-controllable condition.

**Results:**

The study revealed that both reported authorship and controllability scores, as well as the fixation count per second, decreased as μ increased, aligning with trends observed under constant delay conditions. The subjective authorship weakened significantly at a μ of 94 ms. Notably, the comparison between jittery and constant delays indicated the minimum value (μ) of the distribution as a critical parameter influencing both authorship perception and visual search time efficiency.

**Discussion:**

This finding underscores the importance of the shortest delay in modulating SoA. Further examining the relative distribution for fixation duration and saccade amplitude suggests an adaptation in action planning and attention distribution in response to delay. By providing a systematic examination of the statistical attributes of jittery delays that most significantly affect SoA, this research offers valuable implications for the design of efficient, delay-tolerant eye-gaze HCI, expanding our understanding of SoA in technologically mediated interactions. Moreover, our findings highlight the significance of considering both constant and variable delay impacts in HCI usability design, marking a novel contribution to the field.

## 1 Introduction

In everyday life, physical actions are often initiated to achieve objectives. For instance, when entering a hotel building, we stand in front of an automatic door to open the building. As the door opens, it is perceived that *I* stood there, and thus, *I* caused the door to open. This subjective feeling of being the author of one’s actions and their subsequent consequences is referred to as a sense of agency (Gallagher, 2000). However, if the automatic door opened with a significant time delay, one might question whether *I* caused the door to open or if a staff member at the front desk did so instead. The disruption of authorship judgment caused by a temporal discrepancy between one’s action and the resulting external event has been reported in various empirical studies (Sato and Yasuda, 2005; Farrer et al., 2008, 2013; Ebert and Wegner, 2010; Wen et al., 2015a,b; Shibuya et al., 2018).

The *comparator model* has been proposed as an internal framework to explain the sense of agency (Frith et al., 2000; Wolpert and Flanagan, 2001; Blakemore et al., 2002; Haggard and Chambon, 2012). This model posits that when we decide to initiate an action based on intention, we transmit a signal to our muscles while retaining a copy of that signal (efference copy), enabling us to form a prediction. Within the comparator model, we feel a sense of agency over an external event when the afferent signal (signal from the external event) and efferent copies coincide. In contrast, a discrepancy between the efferent and afferent signals suggests that an *other* may be the agent behind the event. The calculated difference between efferent and afferent signals serves not only to negate self-produced sensations (Weiskrantz et al., 1971; Claxton, 1975; Blakemore et al., 1998, 2000) and determine authorship but also to adapt subsequent motor commands for action planning in pursuit of a goal (Haggard, 2017).

Subjective reports and intentional binding have been utilized to explore the sense of agency, particularly regarding the temporal discrepancy between a participant’s action and the subsequent event (Sato and Yasuda, 2005; Farrer et al., 2008, 2013; Wen et al., 2015a; Shibuya et al., 2018; For research implementing both subjective reports and intentional binding, see Ebert and Wegner, 2010; Wen et al., 2015b). Haggard et al. (2002) introduced intentional binding, demonstrating that voluntary actions lead participants to perceive a shorter time interval between pressing a button and hearing a sound compared to involuntary actions. Subjective reports typically involve (1) scoring agreement on event authorship and (2) identifying whether an event corresponds directly to an action (“self”), with a temporal delay (“delay”), or is unrelated (“other”). Generally, the rating score and the frequency of “self” responses decrease with delay, whereas “delay” responses increase. Notably, “other” responses remain rare, even with significant delays, suggesting that temporal delays do not lead to misattribution. This observation challenges the comparator model, which associates the judgment of misattribution with action-outcome mismatches, as noted by Synofzik et al. (2008). They argue that subjective reports capture only the conceptual aspect of the sense of agency (judgment of agency). Furthermore, intentional binding was suggested to address the non-conceptual aspect (feeling of agency) (Ebert and Wegner, 2010; Wen et al., 2015a). By incorporating both questionnaires, our study aims to evaluate the impact of the delay on the sense of agency, acknowledging the inherent limitations of questionnaires yet affirming their utility in assessing delay impact.

Conversely, research on the sense of agency has barely focused on gaze shift as a motor action that exerts control over an eye-tracking device. In particular, the role of action-effect temporal discrepancy as an independent variable has not been fully explored. This study specifically focuses on the sense of authoring control over a gaze-contingent display. Several studies have examined scenarios in which an individual experiences a sense of agency through social gazing, such as when another agent’s gaze follows one’s own, exploring how temporal delays in the other agent’s gaze response affect the sense of agency (Pfeiffer et al., 2012; Recht and Grynszpan, 2019; Brandi et al., 2020). However, the use of temporal delays in the feedback from a gaze-contingent paradigm has barely been studied. While Grynszpan et al. (2012) and Gregori Grgič et al. (2016) demonstrated that eye movements can elicit a sense of agency, the exploration of temporal delays as an independent variable was limited in scope. Gutzeit et al. (2024) investigated the diminishing effect of intentional binding with increasing temporal delays within a gaze-contingent paradigm, yet further research is limited. Nevertheless, findings on the sense of agency related to eye movements are promising, because corollary discharge (or efference copy) from eye movements contributes to visual stability in humans and other animals (Sperry, 1950; von Holst, 1954; Bridgeman, 2010; Cavanaugh et al., 2016). Inactivation of the corollary discharge circuit in monkeys affects visual stability (Cavanaugh et al., 2016). In addition, the efference copy is computed following the comparator model theory (Frith et al., 2000; Wolpert and Flanagan, 2001; Blakemore et al., 2002; Haggard and Chambon, 2012).

The sense of agency is considered crucial for evaluating a user’s experience in human–computer interaction (HCI) (Moore, 2016), emphasizing the user’s feeling of control over the system, rather than being governed by an external agent such as a computer. With the introduction of gaze as a new modality in HCI (Sharma et al., 1998), the significance of the sense of agency in eye-gaze HCI has grown owing to the increasing availability of consumer-level eye trackers such as Tobii (Tobii, n.d.) and the integration of eye movement control in operating systems such as Windows 10 (Microsoft, n.d.). Although eye movements typically serve as tools for social interaction (Emery, 2000) and are seldom used to manipulate the external environment, eye-gaze HCI underscores the importance of studying the sense of agency in relation to eye movements.

Nonetheless, eliminating the temporal discrepancy between a user’s eye movements and the visual feedback of an HCI system is a difficult task. Specifically, in teleoperation scenarios, such as remote surgery (Anvari et al., 2005; Ieiri and Hashizume, 2011) and satellite teleoperation in low earth orbit (Sheridan, 1993; Chen et al., 2022), the delay in capturing a user’s gaze to rendering can reach up to 500 ms. Teleoperation across a network contains the time required for a signal to travel to its destination and return to the local system (i.e., round-trip time, RTT) (Gettys and Nichols, 2012). Furthermore, variations (i.e., inconsistent “jittery” delays) can arise in the RTT for each data point (i.e., packet) because of multiple factors associated with the delivery of packet bits to the destination, such as processing, queuing, transmission, and propagation delays (Roy et al., 2021). While jittery data transmission is known to considerably impact the user experience in video transmission (Claypool and Tanner, 1999; Gulliver and Ghinea, 2007; García et al., 2020), in-depth perceptual studies in teleoperation contexts are scarce. This is attributed to the unpredictable nature of RTTs, which complicates the development of comprehensive models. Factors such as the locations involved in communication, data size, data type, and diverse range of perceptual tasks across scenarios can easily alter the RTTs. Although various models of delay dynamics across regions (Oboe and Fiorini, 1997; Kim et al., 2003; Zhang and He, 2007; Hua et al., 2013; Sukhov et al., 2016) have shown that RTTs typically follow an extremely right-skewed distribution, empirical studies examining user’s performance (Yokokohji et al., 1999; Imaida et al., 2004; Anvari et al., 2005; Ieiri and Hashizume, 2011) and furtherly cognitive workload (Kim et al., 2021; Sasaki, 2022; Musicant et al., 2023; Scholcover and Gillan, 2023; Timman et al., 2023) have not systematically manipulated the variations at RTT. Moreover, owing to scenario-specific limitations, it is challenging to generalize these findings to a perceptual context concerning the sense of agency and temporal discrepancy. When considering the internal mismatches (e.g., delay) computation in the comparator model, our interest is aroused when such a computation is disrupted by jitter. What is the calculated value of the prediction errors that we can access or become aware of? Is it the minimum, maximum, or mean value of the jittery distribution?

This study introduced action-effect jittery temporal delays and eye-gaze HCI to investigate the sense of agency. Despite the limitations of subjective reports, such as susceptibility to biases and their criticism of only capturing the conceptual judgment of agency, we believe they offer value by enabling direct comparisons with prior research (Farrer et al., 2008, 2013; Kim and Yoshida, 2023). Additionally, this study sought to examine the impact on the participant’s eye movements behavior, enabling us to propose altered behavior patterns as an indicator of a disrupted user experience. Furthermore, our goal was to identify the statistical attributes within the distribution of jittery delays that best account for the effect on the sense of agency and eye movements (i.e., minimum, mean, or maximum value of the distribution).

Overall, this study was guided by the following research questions:

1. Does a jittery delay influence the sense of agency in relation to eye movement?
2. How does temporal discrepancy affect eye movement behavior?
3. Are there specific statistical attributes that most effectively explain the sense of agency and eye movements with a jittery delay?

## 2 Materials and methods

**Figure 1:**
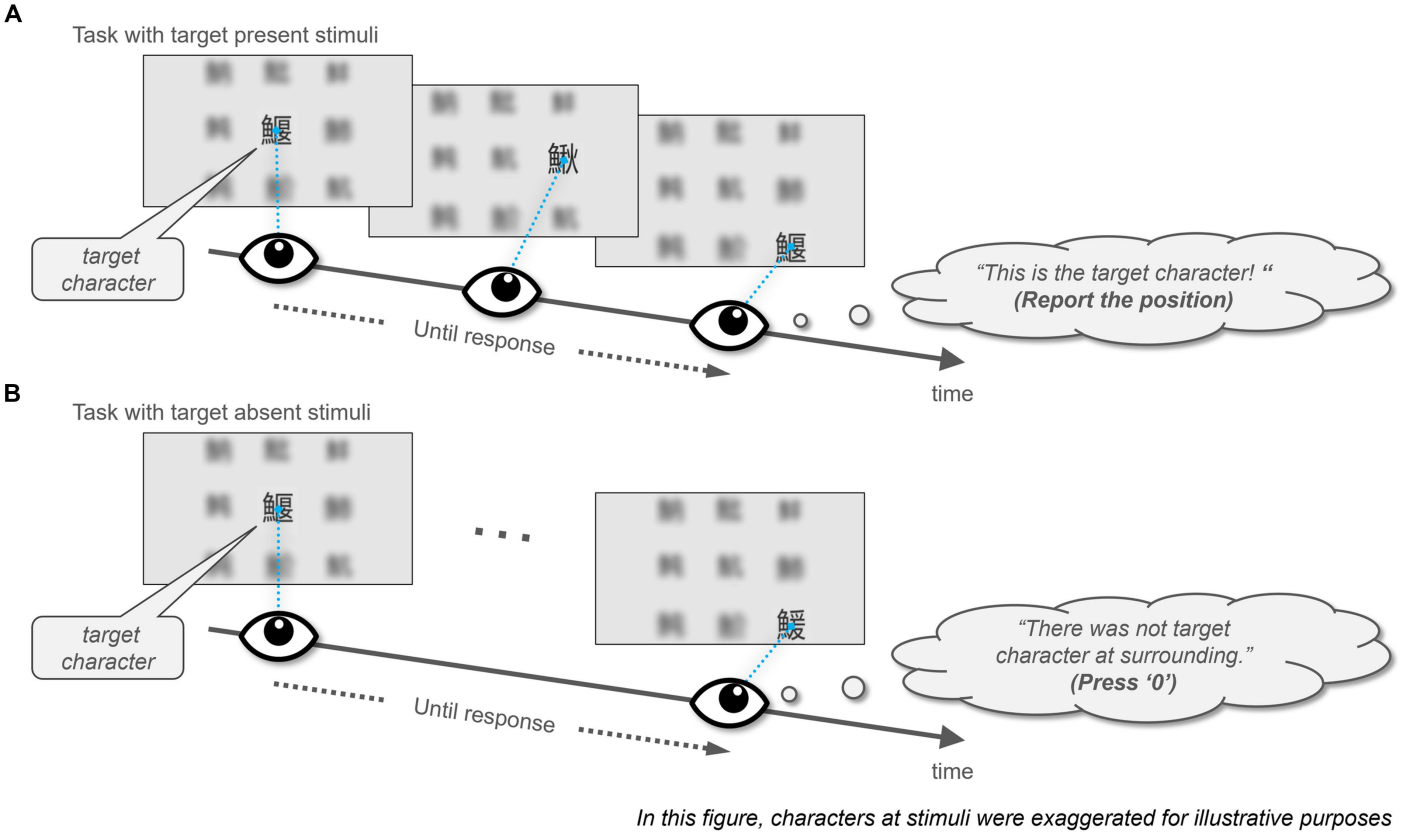
Illustration of the task in the experiment. Participants conducted a visual search for Chinese characters using a biresolution, gaze-contingent display. This display rendered a 3 × 3 array of Chinese characters in high resolution within the participant’s central vision and a blurred version in the peripheral view. Characters in this figure are intentionally magnified for illustrative purposes. Comprehensive details about the stimuli are provided in Section 2.3. The participant’s task was to locate a target character that matched the central character within the eight surrounding characters, which may have been absent or uniquely present in the surrounding locations. **(A)** Task with target present stimuli. **(B)** Task with target absent stimuli.

In this study, the participants were required to conduct visual search tasks using a bi-resolution gaze-contingent display that limited the area of the high-resolution display to a square shape in the participant’s central field of view (for a detailed description of the stimuli used, refer to section 2.3 Stimuli). We presented stimuli in two distinct regions with an unblended border: the stimuli consisted of a 3 × 3 Chinese character array rendered within the high-resolution window and a blurred array in the surrounding area. Participants received instructions to find a specific character, identical to the one displayed at the center, among eight surrounding positions (Figure 1). This target character might not be present or could appear just once within the given stimuli. After each task, participants were asked to answer the two questionnaires.

Fifteen sessions (270 trials) were conducted for each participant. Each session comprised 18 trials: two practice trials, two repetitions of seven delayed trials, and two uncontrollable playback trials. In practice trials, the gaze-contingent window was updated without an intentional delay. In delayed trials, the window had a temporal delay between the participant’s eye movement and the display update, which followed a truncated normal distribution. The uncontrollable playback trial was set to assess whether our questionnaires could capture the participants’ misattribution. The playback trial involved our gaze-contingent display replaying the participant’s gaze behavior from one of the practice trials rather than mirroring their real-time gaze. Following the two practice trials, delayed and playback trials were presented randomly.

**Figure 2:**
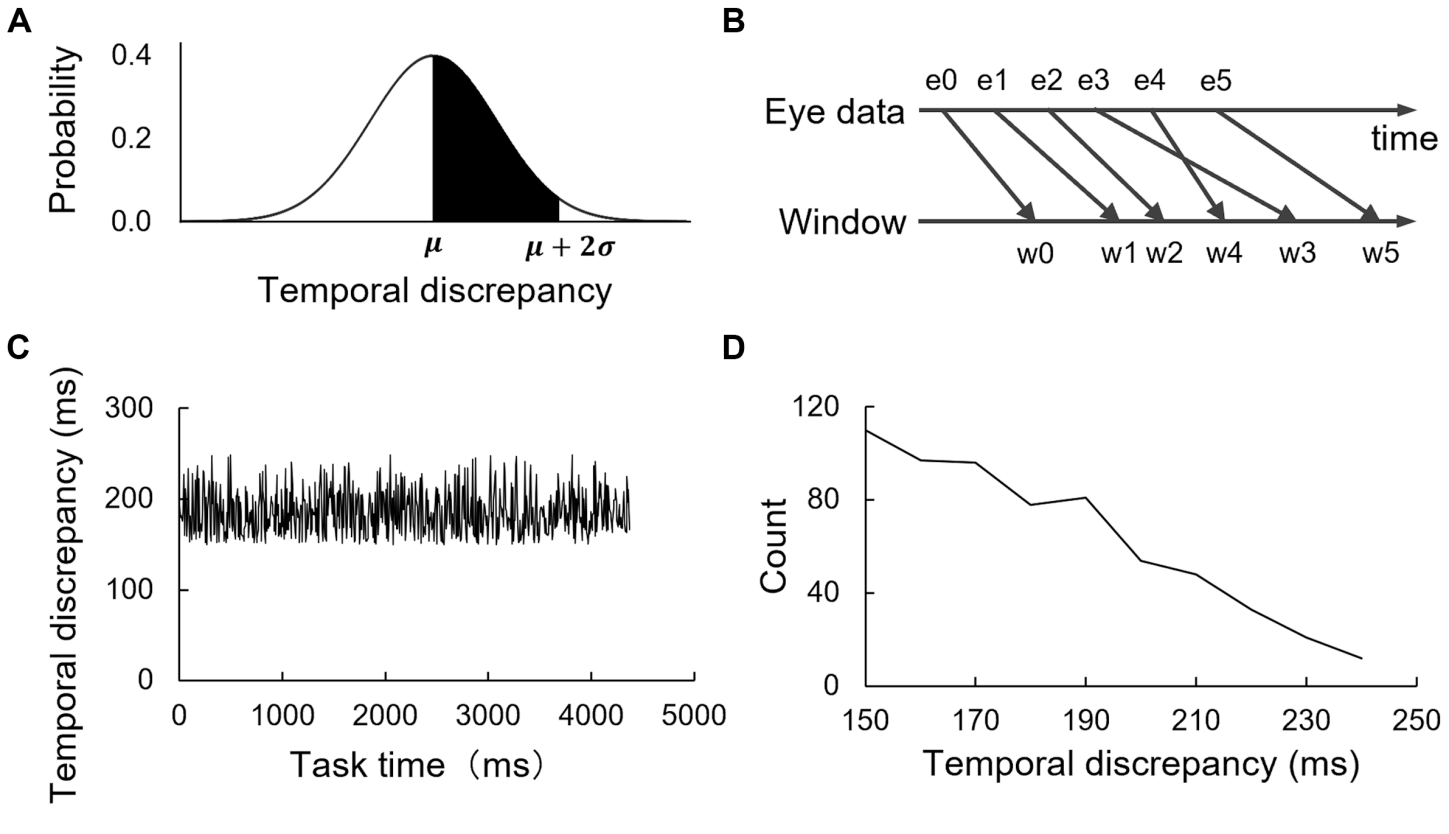
Illustration of jittery delays and their distribution in the gaze-contingent display. (**A**, top-left) The temporal discrepancy between the recording of eye movements and the updating of gaze-contingent display was randomly adjusted for each eye movement data point within a range from 𝜇 to μ + 2σ, following a normal distribution. (**B**, top-right) Distinct data points had different delays, occasionally causing a reverse-order display. A sample trial featuring a jittery delay is illustrated in (**C**, bottom-left) as a time function and (**D**, bottom-right) as a distribution (𝜇 = 150, 𝜎 = 50) with a 10 ms bin. A representative value of each bin placed at its minimum (e.g., the value of a 240–250 ms bin is shown at 240 ms).

We set σ to 50 ms and established seven conditions for 𝜇: 0, 50, 100, 150, 200, 300, and 400 ms with a truncated normal distribution (from 𝜇 to μ + 2σ where 𝜇 is mean and σ is standard deviation, Figure 2A) for the jittery delay. In a trial, the temporal discrepancy was randomly generated for each eye movement data point. Due to its random generation properties, the gaze-contingent window occasionally displayed eye data in reverse order (w3 and w4 in Figure 2B). A sample of the randomly generated temporal discrepancy throughout a trial is depicted in Figure 2C as a time function and Figure 2D as a distribution (𝜇 = 150, 𝜎 = 50).

Prior research suggested that RTTs generally followed highly right-skewed distributions such as gamma (Kim et al., 2003; Hua et al., 2013), exponential (Oboe and Fiorini, 1997; Sukhov et al., 2016), or Pareto (Zhang and He, 2007) distributions. Our goal was to investigate each statistical aspect of jittery delay (minimum, mean, and maximum) and to emphasize the differences among these aspects. While highly right-skewed distributions often exhibit similar minimum and mean values, the truncated normal distribution provides defined boundaries, aiding in the differentiation between the statistical aspects of jittery delay. Hence, we adopted this distribution in the current study. The delay ranges were set to adequately cover the areas where subjective authorship reports previously showed a decrease, while also realistically representing teleoperation scenarios.

Participants reported the extent to which they felt that their eye movements caused the window to move, using a subjective rating score on a six-point scale, a method commonly employed in previous research (Sato and Yasuda, 2005; Ebert and Wegner, 2010; Wen et al., 2015a,b; Shibuya et al., 2018). Additionally, this study incorporates a questionnaire from Farrer et al. (2008) to investigate the influence of the action-effect temporal delay on authorship across three levels: perfect control, imperfect control, and control by another agent, as described by Farrer et al. (2013).

We designed the visual stimuli according to the concern of decreasing saccade amplitude when the peripheral field contains less spatial information than the central field of view with the gaze-contingent display similar to ours (Reingold et al., 2003; Foulsham et al., 2011; Laubrock et al., 2013; Cajar et al., 2016a,b). This observation is made in comparison with the presentation of a typical uni-resolution display. Previous studies have suggested that reduced saliency beyond the window and comparatively heightened saliency within the window can influence eye movements. To address this concern, we organized nine black Chinese characters in a three-by-three array, aiming to maintain the saliency of the blurred periphery in contrast to the background.

The participants also completed two sessions (36 trials each) of the visual search task without a gaze-contingent display. Under this condition, the participants were not required to answer any subjective questionnaires. This condition aimed to examine the impact of the gaze-contingent window on eye movements and task difficulty. In each session, the participants were shown 36 random images selected from a pool of 540 images.

### 2.1 Participants

A total of 12 individuals were enlisted in the study; however, data from one individual were excluded because of technical problems in registering keyboard responses. Therefore, this study analyzed 11 individuals who were all students (six undergraduates, three pursuing master’s degrees, and two enrolled in doctoral programs) at the Tokyo Institute of Technology. Two participants did not participate under the no-window condition. The participants had an average age of 23.9 years, with a standard deviation of 2.4 and an age range of 19–27 years. Among the participants, two were women and nine were men, including three Japanese, six Korean, and two Chinese. This study was announced to undergraduate students during their classes, and master’s and doctoral program students were informed through their advisors via email. Only participants who could read Chinese characters were recruited as we used characters with a consistent radical character (please refer to the section on stimuli). Each hour of participation in the experiment earned the participants a 1,000-yen Amazon gift card. All participants had normal or corrected-to-normal vision and provided informed consent before participation. The experiments were conducted in accordance with the guidelines of the Declaration of Helsinki. An institutional consent form concerning consent to publish was obtained from all study participants. The Tokyo Institute of Technology Ethics Review Committee for Epidemiological Research approved this study. Data were collected between April 26, 2022, and December 15, 2022. Subsequent to this period, statistical analyses and evaluations were performed to address the research questions.

### 2.2 Apparatus

**Figure 3:**
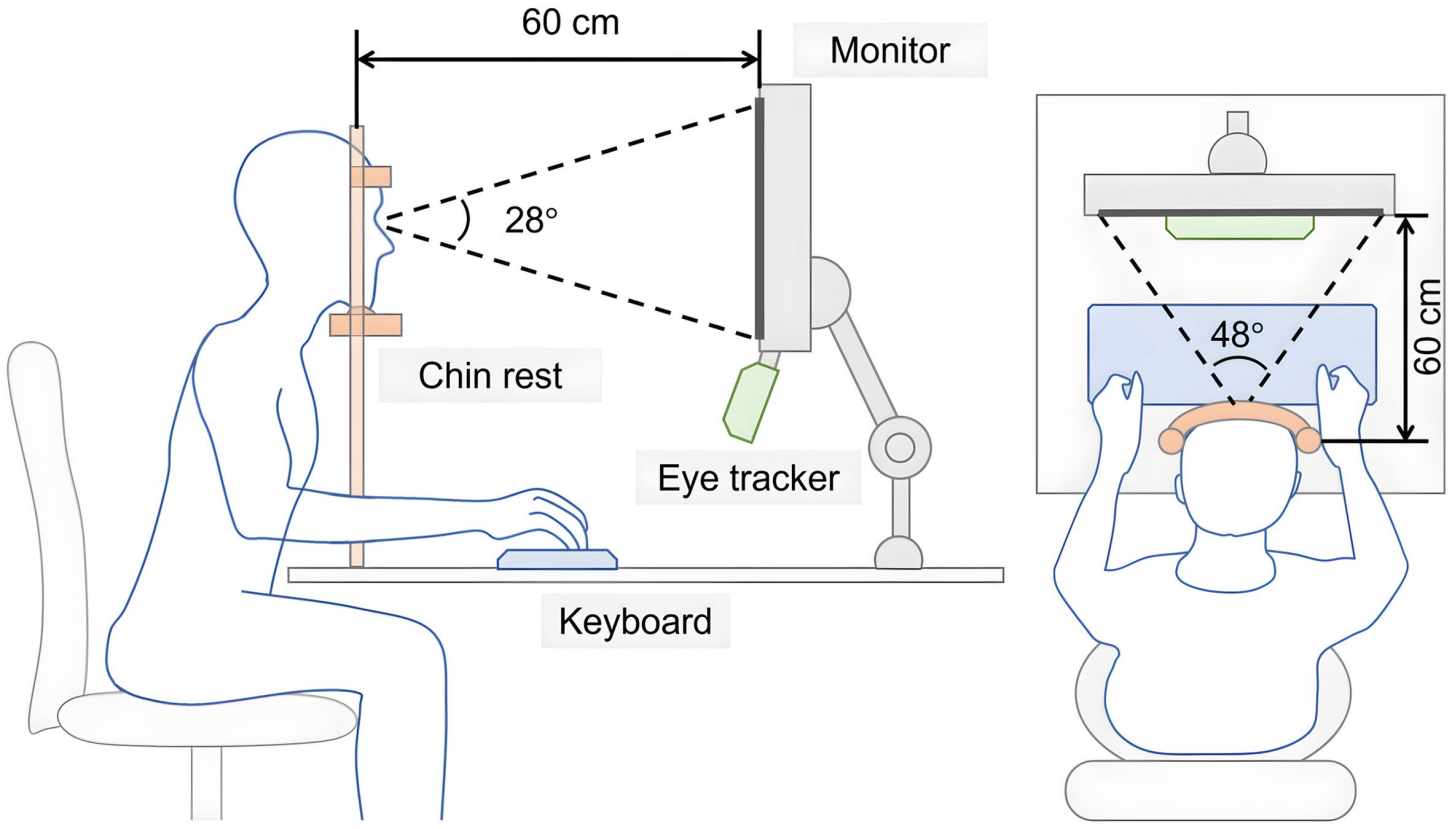
Experimental setup. The experiment took place in a dimly lit room where visual stimuli were displayed on an LCD screen. To reduce head movement, participants placed their chin and forehead steadily on a chin rest. Responses were registered using a numerical keypad attached to a keyboard, and an eye-tracking device captured the gaze data from the participant’s left eye.

Figure 3 illustrates the experimental setup. The participants completed the experiment in a dark room where they were presented with visual stimuli through an LCD (24 inches, XL2411t, BenQ Corp., Taipei, Taiwan, frame rate:144 Hz; viewing distance:60 cm). To minimize head movement, the participants were instructed to position their chin and forehead on a chin rest. The delay from eye movement to the monitor’s feedback was determined by the eye tracker’s sample delay (mean < 1.4 ms, SD < 0.4 ms), processing time (updated every 1 ms), and input lag on the display. Participants responded using a numeric keypad on a keyboard (SK-8825; Lenovo Group Ltd., Beijing, China). The EyeLink 1000 Plus (SR Research Ltd., Ottawa, Ontario, Canada) recorded the participant’s left-eye gaze with a spatial resolution of <0.02° and a sampling rate of 2,000 Hz. Calibration and validation were conducted using a nine-point procedure with a validated accuracy of <1°. After each session, participants took a break and underwent recalibration and validation. The software program for the experiment was modified from the GCWINDOW template provided by SR Research, Ltd.

### 2.3 Stimuli

**Figure 4:**
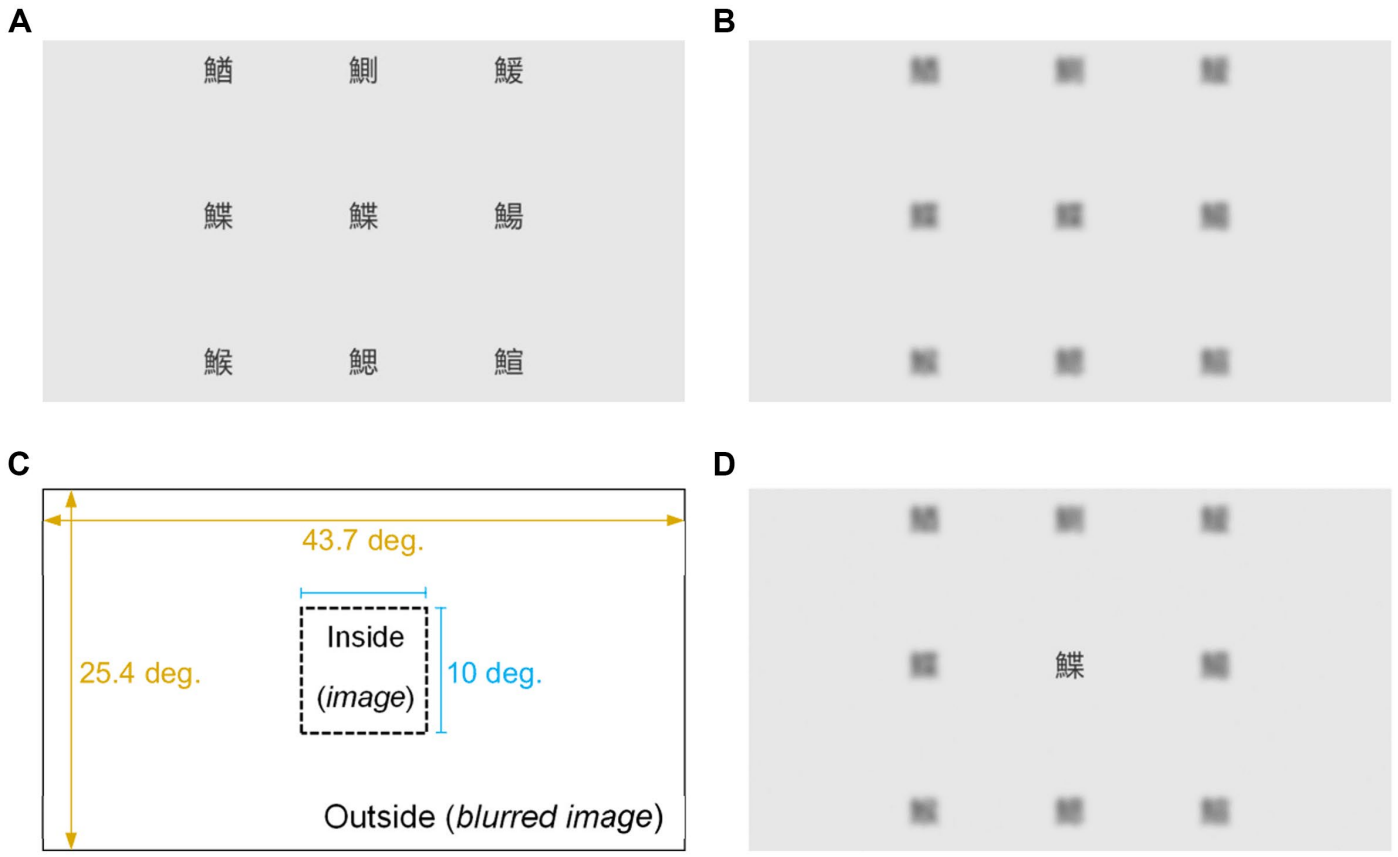
Stimulus design. Representation of the stimulus and gaze-contingent display used in the experiments. (**A**, top-left) A stimulus featuring nine Chinese characters arranged in a three-by-three virtual matrix is generated randomly. All nine characters shared the same stroke count and had a fish radical. A total of 540 images were generated, with the target character either present or absent. (**B**, top-right) One of the 540 generated images was randomly chosen and blurred. (**C**, bottom-left) Inside the gaze-contingent display (rectangle with solid lines), a virtual gaze-contingent window (square with dashed lines) was positioned at the center of the participant’s fixation location. An image was displayed within the window, while the blurred image surrounded the central area. (**D**, bottom-right) An example of the stimulus is when the participant focused their gaze on the central Chinese character.

In the visual search array, the characters were black (luminance: 4.71 ± 1.32 $cd/m^{2}$ ) against a gray background (luminance: 30.41 ± 3.15 $cd/m^{2}$ ). Each character was 3° in size, with horizontal and vertical distances of 11° between adjacent characters (Figure 4A). Blurred image (Figure 4B) was produced by applying a Gaussian filter to one image using Adobe Photoshop 2020. A low-pass filter with a standard deviation of 10 pixels (equivalent to 0.2°) is implemented. The signal was reduced by 3 dB at a spatial frequency of 0.57 cycles/°, and the attenuation became stronger at higher spatial frequencies. By applying a Gaussian filter, the luminance of the blurred Chinese characters increased (15.27 ± 3.76 $cd/m^{2}$ ), while the gray background’s luminance remained consistent.

A dynamic 10° square gaze-contingent window was centered on participants’ fixation locations and adjusted according to their eye movements (Figure 4C). The edge of the window was 5° vertically or horizontally from the fixation location. The image (Figure 4A) appeared only within the central moving window, whereas a blurred image (Figure 4B) was displayed in the surrounding external area (Figure 4C). The image was changed from the inner to the outer side of the square window using a step function. An example rendered image is shown in Figure 4D. Despite designing the display to allow reading of only one character through direct fixation, participants could adjust their gaze strategically to view two characters simultaneously. Nonetheless, our analysis suggests that such a strategy was not utilized by the participants (refer to Supplementary material S1 for details).

Nine Chinese characters were randomly arranged in an array according to the following three rules: First, each character had a Chinese character “fish” as a radical character, ensuring all characters belonged to the same meaning category (fish) to avoid meaning-or category-based attentional bias. Second, the characters had the same stroke count to ensure consistent spatial frequency, contrast, and complexity. Third, the central character matched one of the other eight characters with a 50% probability (target or non-target). Such uncertainty was implemented to prevent participants from guessing the target’s location before completing the visual search, which could influence their behavior. The minimum correct response rate for the tasks was 98%, demonstrating that participants actively participated in each trial without skipping (the worst error rate per individual was 2.1%; see Supplementary material S2 for details). A total of 239 Chinese characters (see Supplementary material S3) met the first two criteria. A total of 540 image files were created using preprogrammed random image-generating Unity software. We coded the software to specify the existence and location of the target using a random number generator function. As we did not sample the stimuli, the target existence and location probabilities were uneven. Of the 540 images, 272 were no-target arrays and 268 were target arrays, with varying target counts at each of the following eight locations (clockwise from the middle top): 34, 29, 32, 32, 35, 40, 37, and 29.

### 2.4 Procedure

**Figure 5:**
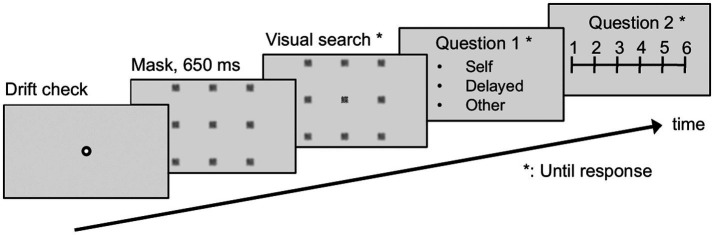
Trial sequence in the experiment: Following the drift check, participants waited for the gaze-contingent window to appear. They could only read a Chinese character within the virtual gaze-contingent window. After responding, participants completed two questionnaires. The drift check and questionnaires were enlarged for clarity in the explanation.

As shown in Figure 5, each trial commenced with a drift check, followed by a 650 ms display of blurred images, before initiating the visual search. This predetermined duration, which was consistent across all trials, prevented participants from expecting any deliberate temporal discrepancies. During the 650 ms waiting period, the participants were instructed to fixate on the center of the monitor. The visual search concluded when the participants identified and reported the target’s location and pressed the enter key. Subsequently, they completed the authorship questionnaire to assess whether they perceived the window movements as aligned with their gaze (self), temporally delayed (delay), or controlled by another agent (other). They then rated the degree to which they believed their eye movements influenced the window’s movement on a six-point scale, where six represented “I fully manipulated the window” and one indicated “I could not possibly manipulate the window”—this was the authorship rating questionnaire.

### 2.5 Analyses

All statistical analyses were performed on the average values for each condition, representative of each participant.

To investigate the delay’s impact on the sense of agency, our study’s first question, we utilized two generalized linear mixed models (GLMM; SPSS 24.0) to analyze the 𝜇 value’s effect on categorical authorship reports. Specifically, for trials involving delays—excluding playback and no-delay trials—we compared “self” to “delay” responses and “other” to “delay” responses, using a multinomial probability distribution and a generalized logit link function. The participant served as a random effect, with variance components as the covariance structure, to capture unexplained variance, with 𝜇 value treated as a fixed effect. Due to data loss, four responses were excluded, resulting in 2,306 responses analyzed. For mean authorship rating scores, we conducted a one-way ANOVA with repeated measures on delayed trials. Mauchly’s test assessed sphericity, with degrees of freedom adjusted using Greenhouse–Geisser estimates.

To address our second question on the impact on participants’ behavior, we first analyzed response times. Response time was assessed as a measure of task complexity. The duration from stimulus onset to the registration of the participants’ final input was recorded. Previous inputs were disregarded as repeated registrations frequently occurred when the participants needed to reposition their hands on a keypad. We conducted a target × delay repeated-measures ANOVA on the response time under the delayed conditions. The same procedure was applied to adjust degrees of freedom. *Post-hoc* analyses were conducted using Bonferroni correction for pairwise comparisons. For eye movements, we examined fixation counts per trial, fixation counts per second, relative distribution for fixation duration, and saccade amplitude, all in relation to the 𝜇 value. we set the minimum thresholds for eye movements to 0.1°, 30.0°/s, and 8000.0°/s^2^ to identify saccades. Any fixations that occurred outside the stimuli, began immediately before or after a blink, or lasted less than 120 ms were excluded from our analyses. We determined the relative distribution of fixation durations in 25 ms intervals within a 0–800 ms range and the distribution of saccade amplitudes in 0.5° intervals within a 0–15° range. Given the variability in response times across trials and participants, we opted for relative distributions. For the fixation counts per trial, we conducted a one-way (delay) repeated-measures ANOVA under the delayed conditions. Mauchly’s test assessed sphericity, with degrees of freedom adjusted using Greenhouse–Geisser estimates.

Our third aim was to identify the statistical attributes (e.g., minimum, mean, or maximum values of the distribution) that had the greatest influence on the sense of agency and eye movements. We compared our present results (i.e., the categorical authorship report and fixation count per second) with those of our previous experiments, in which a constant delay ranging from 0 to 4,000 ms was implemented (Kim and Yoshida, 2023). In the previous study, the same gaze-contingent window, as well as the no-window and playback conditions, was applied as in the current study. For comparison, we utilized data from delayed conditions ranging from 0 to 400 ms, which were retrieved from our previous publication on the open science framework (more details on the methods and results from the previous experiment can be found in Supplementary material S4). We only considered the mean (𝜇) value in the jittery distribution implemented in the current study, which represents the minimum value of that jittery distribution. If the comparison did not reveal a significant difference between the two experiments, we concluded that the minimum value was the most influential attribute.

With fixation count per second, we conducted two-way mixed ANOVA for the five delayed trials (0, 100, 200, 300, and 400) with delay as a within-subjects factor, and experiment type (constant and jittery) as a between-subjects factor. With categorical authorship reports, using the least-squares method, we fit a sigmoid function to the proportion of each of the two categories in the authorship report (self and delay). The *other* responses were omitted, as they were barely reported across both experiments. For each experiment, we fit two regression curves for each participant, yielding 38 curves. The sigmoid function $Y=1/\left(1+e^{a\left(x-b\right)}\right)$ was defined, with a and b as its two adjustable parameters. The b indicates the delay at which the response proportion reaches the 50% threshold, whereas a indicates the slope of the threshold. A positive value of a represents an increasing slope, whereas the opposite is true for a negative value. Removing a fit with a $R^{2}$ less than 0.70 resulted in the exclusion of one delay response curve from the current jittery delay experiment, leaving a total of 37 curves for comparison. We chose to calculate the 50% threshold, which is considered a reliable statistical measure of the curve (Sato, 2008; Shimada et al., 2010; Farrer et al., 2013) as it represents the alteration in the subjective sense of agency. Moreover, the slopes reflected the degree of uncertainty in the participants’ responses, with steeper slopes indicating higher certainty. Consequently, we fitted a sigmoid function as previously described by Farrer et al. (2013). With fitted slope values ( a ) and threshold delay values ( b ), one-way multivariate ANOVA was conducted for the delayed trials with the experiment type as a between-subjects factor.

## 3 Results

### 3.1 Subjective authorship

**Figure 6:**
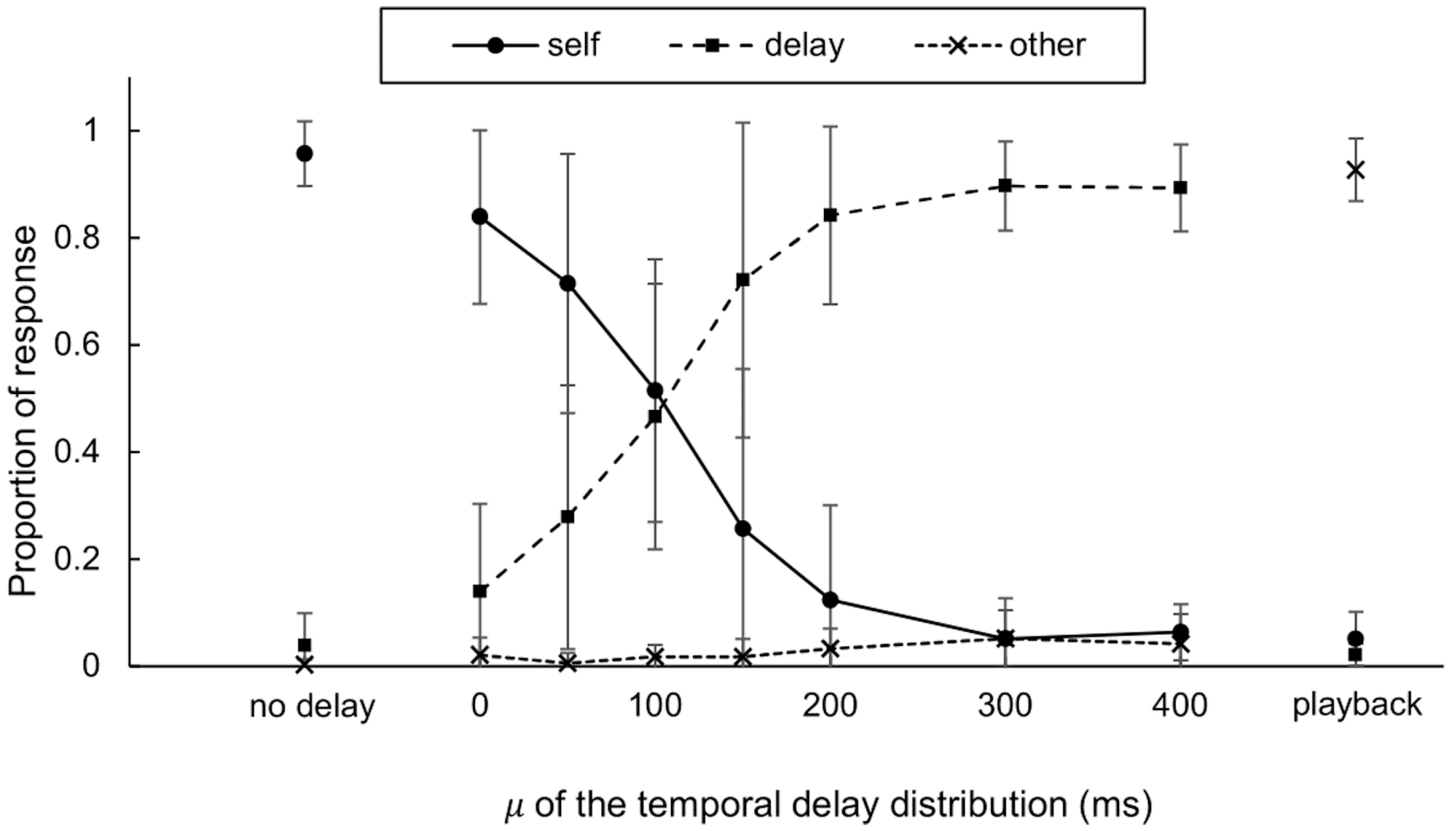
The proportion of participant responses on categorical authorship (self, delay, or other). Means and standard deviations were computed among the participants (*N* = 11).

The categorical authorship report includes three levels of responses (self, delay, and other). For each condition, we computed the mean and standard deviation of the proportion of each response type, as shown in Figure 6. The GLMM results revealed a significant main effect of delay in the comparison between “self” and “delay” responses (*b* = −0.018, *SE* = 0.002, *t* = −8.913, *p* = 0.000), indicating a less “self” than the “delay” responses with increasing 𝜇. All parameters and coefficients for the GLMM are available in Supplementary Table S4. When comparing the “other” response to the “delay” response, the main effect of the delay was not significant (*b* = 0.000, *SE* = 0.002, *t* = 0.122, *p* = 0.903) while only the intercept showed significance (*b* = −3.387, *SE* = 0.507, *t* = −6.683, *p* = 0.000), indicating the infrequent choice of “other” response in delay condition. All parameters and coefficients for the GLMM can be found in Supplementary Table S5.

**Figure 7:**
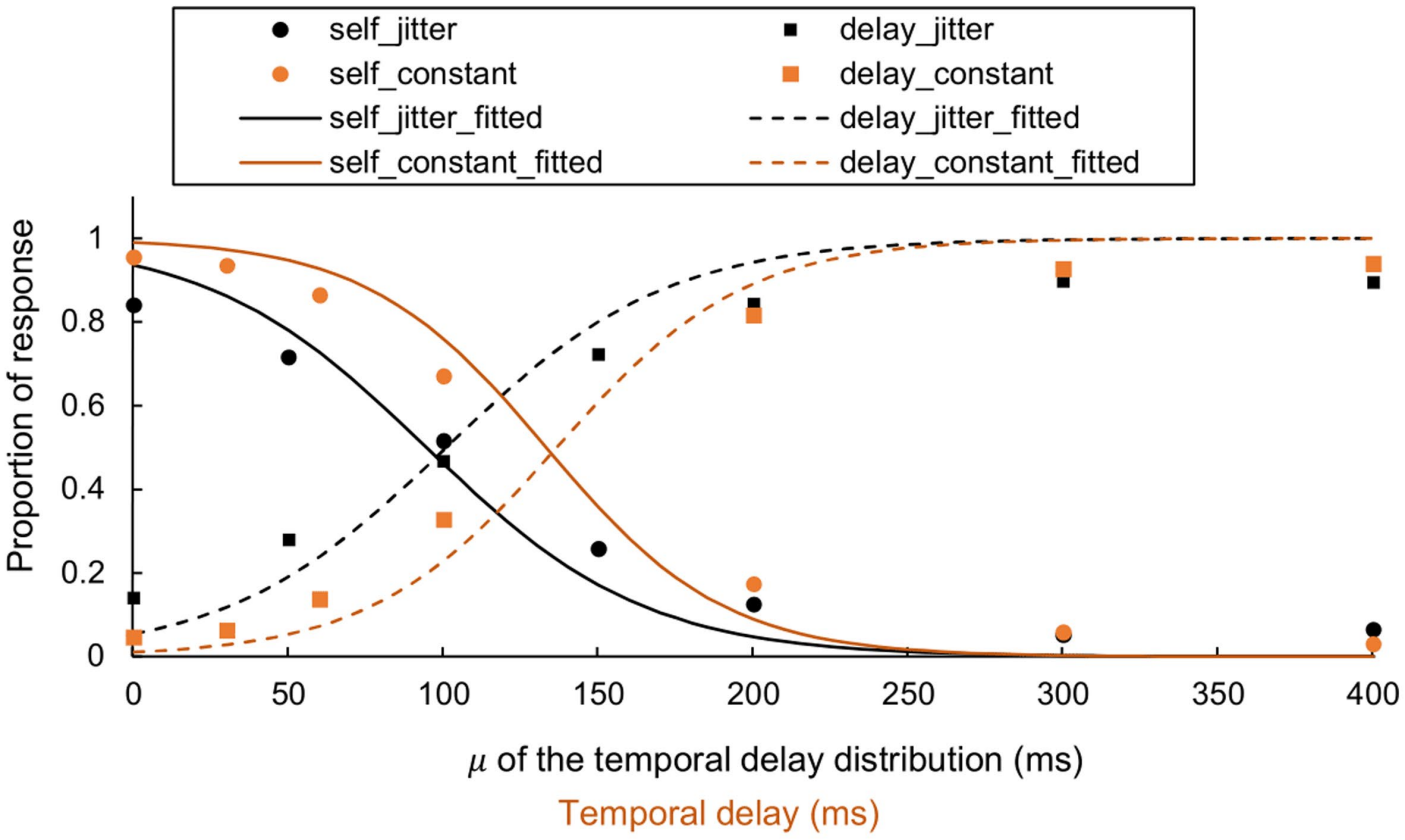
Comparison of categorical authorship responses and fitted function between jitter and constant delays. The fitted sigmoid function illustrates the reported authorship (self vs. delay) for both the current experiment (jitter condition, *N* = 11) and the previous study (constant condition, *N* = 8, Kim and Yoshida, 2023). For visualization, data points from all participants are combined, representing a unified measure of the reported authorship. Each analysis (i.e., curve fitting), was conducted for every participant and condition. Note that the “self_jitter” and “delay_jitter” correspond to the average proportion of the “self” and “delay” responses in Figure 4.

The means and standard deviations for each fitted slope value ( a ) and threshold delay value ( b ), across each categorical authorship response in both previous (Kim and Yoshida, 2023) and current experiments, are detailed in Table 1 (for a visualized graph, see Figure 7). Note that the “self_jitter” and “delay_jitter” in Figure 7 correspond to the average proportion of the “self” and “delay” responses in Figure 6. The multivariate ANOVA, assessing the impact of experiment type (constant vs. jittery), did not yield a significant main effect (*F* [4, 13] = 0.459, *p* = 0.764, partial $\eta^{2}$ = 0.124). All parameters for the MANOVA are available in Supplementary Table S6.

**Table 1:** Fitted parameters on two categorical authorship responses (self and delay) between jitter and constant delays.

Study	Response	Parameter	Mean	Standard deviation
Current study (jittery delay)	Self	a	0.0284	0.0104
		b (ms)	94.34	66.98
	Delay	a	−0.0283	0.0114
		b (ms)	100.74	69.02
Previous study (constant delay)	Self	a	0.0347	0.0133
		b (ms)	132.97	51.35
	Delay	a	−0.0332	0.0141
		b (ms)	136.42	54.36

Mean and standard deviations for each fitted parameter at each response (self and delay) for the current study and previous study (Kim and Yoshida, 2023). The b indicates the delay value where the response proportion reaches the 50% threshold. The a indicates the slope at the threshold value (positive a for the increasing slope and the opposite for the decreasing slope).

**Figure 8:**
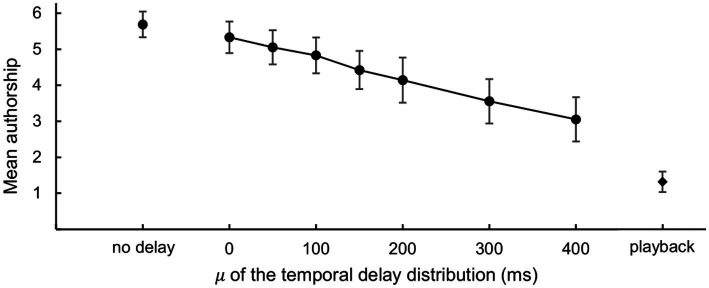
Authorship ratings by participants. The mean authorship ratings and standard deviations were calculated among the participants (*N* = 11).

The authorship rating with a scale of one to six gages the subjective authorship, where one implied “I could not possibly manipulate the window” and six represented “I fully manipulated the window.” The averages and standard deviations of the ratings were calculated for each participant (Figure 8). Mauchly’s test indicated that the assumption of sphericity had been violated ( $\chi^{2}$ [20] = 99.822, *p* = 0.000). Therefore degrees of freedom were corrected using Greenhouse–Geisser estimates of sphericity (ε = 0.217). The main effect of the delay was significant (*F* [1.303, 13.025] = 65.974, *p* = 0.000, partial $\eta^{2}$ = 0.868), suggesting that delay affects the subjective authorship of the gaze-contingent window. All parameters for the ANOVA are available in Supplementary Table S7.

### 3.2 Behaviors

**Figure 9:**
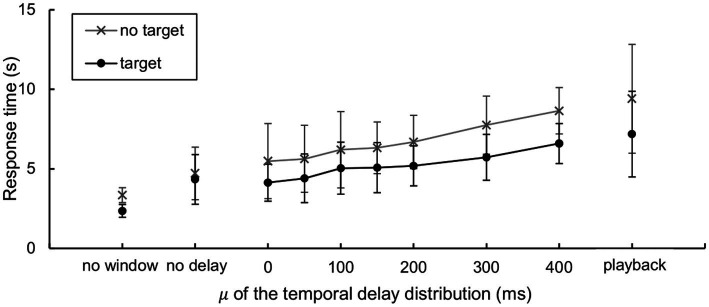
Participants’ response times. Means and standard deviations of the response time (*N* = 11). Note that the two participants did not conduct the “no window” condition.

Both the mean and standard deviation of the response times were calculated based on each participant’s average response time under each condition and in the presence or absence of the target (Figure 9). Mauchly’s test indicated that the assumption of sphericity had been violated for the delay ( $\chi^{2}$ [20] = 44.342, *p* = 0.002) and the interaction ( $\chi^{2}$ [20] = 50.915, *p* = 0.000). Therefore degrees of freedom were corrected using Greenhouse–Geisser estimates of sphericity (ε = 0.289 for delay, ε = 0.399 for the interaction). The main effect was significant for the delay (*F* [1.734, 17.341] = 33.814, *p* = 0.000, partial $\eta^{2}$ = 0.772) and target (*F* [1, 10] = 23.471, *p* = 0.001, partial $\eta^{2}$ = 0.701), whereas the interaction was not significant. These results indicate that the response time is affected by temporal delay. All parameters for the ANOVA are available in Supplementary Table S8. A *post hoc* pairwise comparison with Bonferroni correction found that the response time significantly increased by a mean difference of 1,511 ms (*p* = 0.001) when the target-absent condition (*M* = 6,674 ms) was compared to the target-present condition (*M* = 5,163 ms), suggesting that our gaze-contingent display forces a visual search in a serial manner.

**Figure 10:**
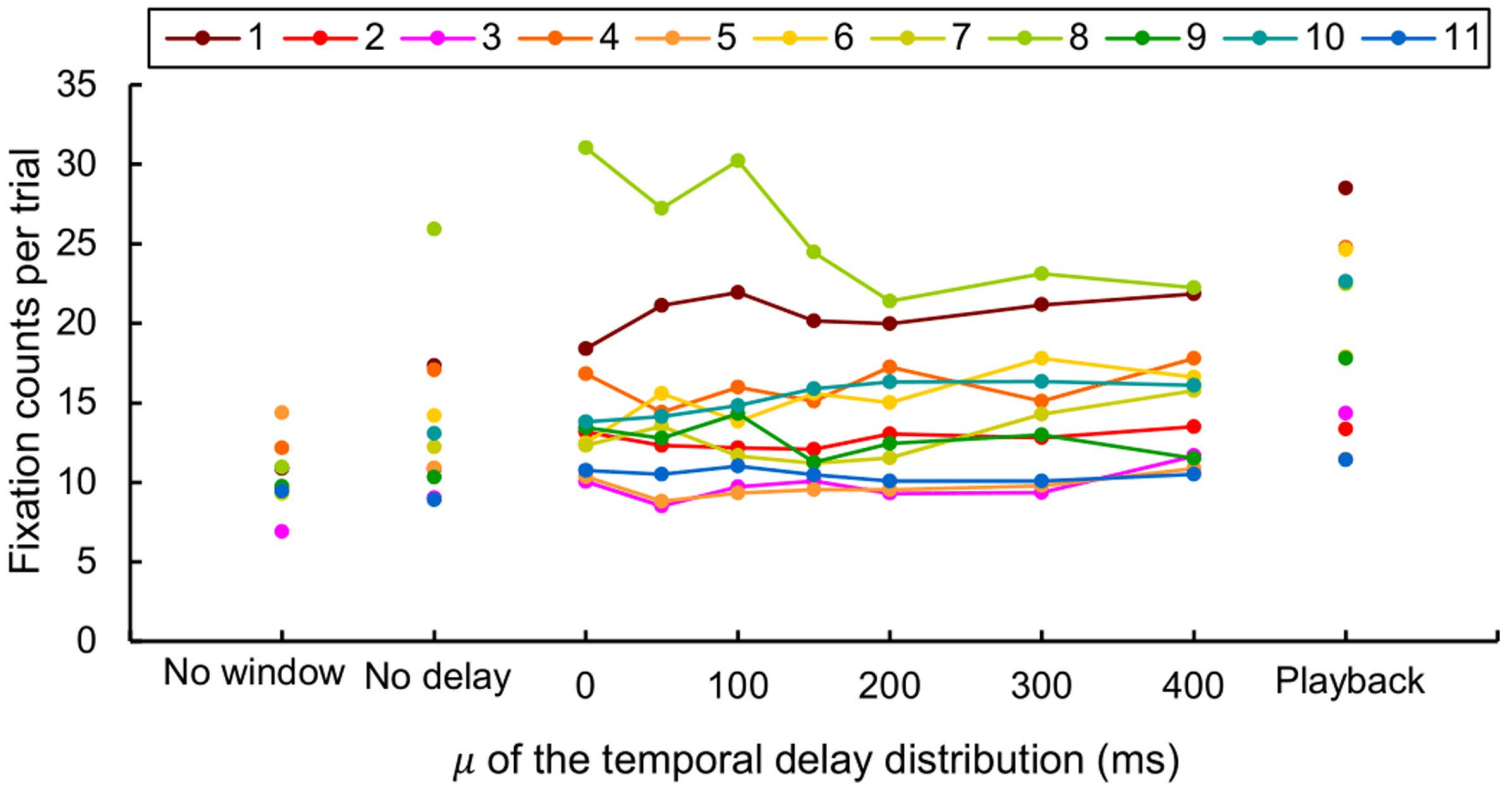
Fixation counts per trial for each of the 11 participants. Note that the two participants did not conduct the “no window” condition.

The fixation counts per trial were presented per participant (Figure 10) as the counts largely varied across participants. Mauchly’s test indicated a sphericity assumption violation for the delay ( $\chi^{2}$ [20] = 53.481, *p* = 0.000), hence the Greenhouse–Geisser estimates were used to adjust degrees of freedom (ε = 0.292). Delay did not yield a significant main effect (*F* [1.751, 17.512] = 0.774, *p* = 0.460, partial $\eta^{2}$ = 0.072). All parameters for the ANOVA are available in Supplementary Table S9.

**Figure 11:**
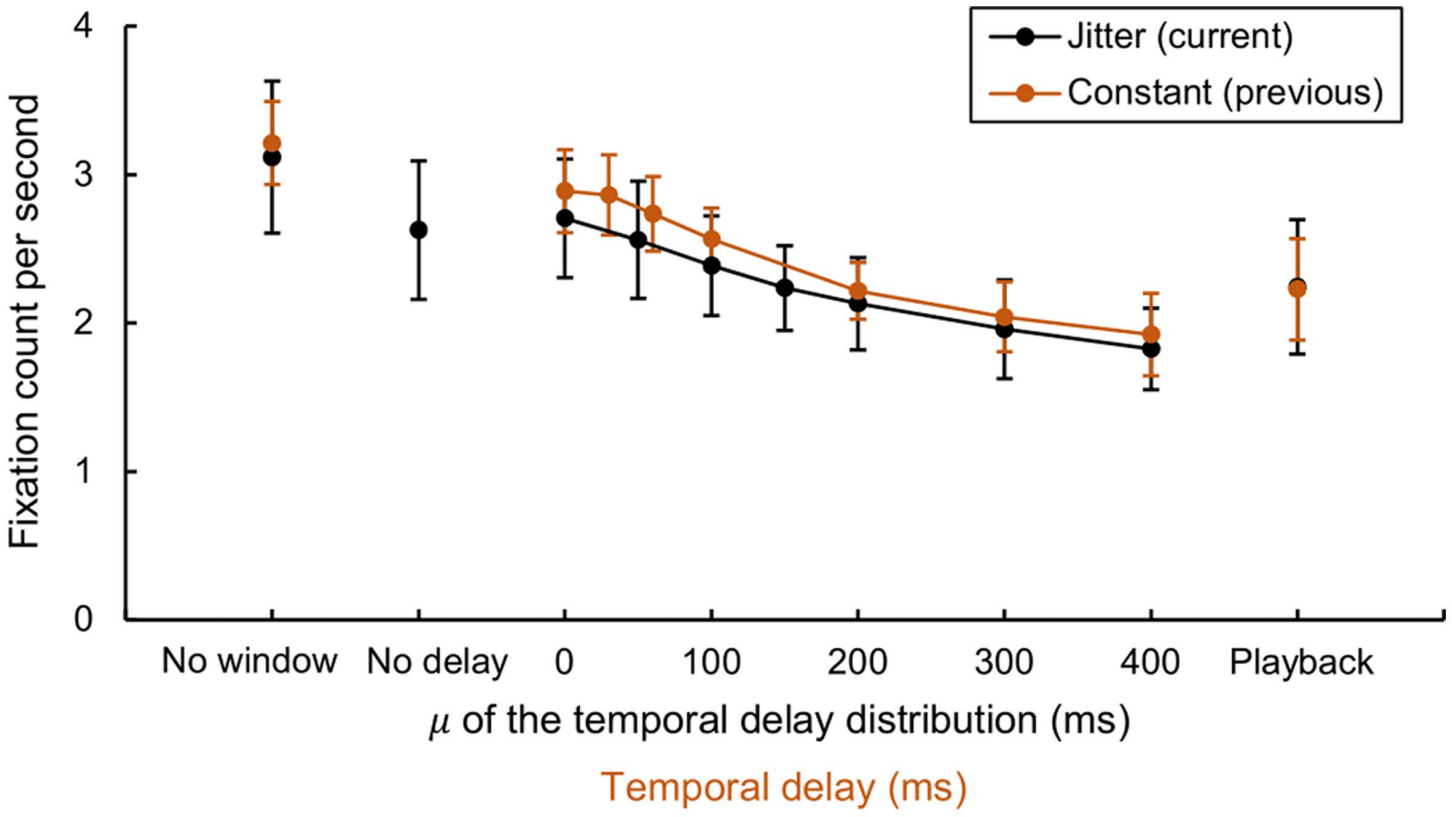
Comparison of fixation counts per second between jitter and constant delays. Mean fixation count per second for both the current experiment (jitter delay, *N* = 11) and the previous study (constant delay, *N* = 8, Kim and Yoshida, 2023). Mean values among participants were displayed with standard deviation as the error bars. Note that the “no delay” condition in the current experiment is virtually identical to the “0 ms” condition in the previous experiment in that no intentional delay was introduced.

The mean fixation count per second and its standard deviations were calculated for both the current experiment (jitter delay, *N* = 11) and the previous study (constant delay, *N* = 8, Kim and Yoshida, 2023) (Figure 11). The experiment type did not yield a significant main effect (*F* [1, 17] = 0.961, *p* = 0.341, partial $\eta^{2}$ = 0.053). Delay showed a significant main effect in both experiments (*F* [1.473, 25.034] = 90.652, *p* = 0.000, partial $\eta^{2}$ = 0.842) where Mauchly’s test indicated that the sphericity violation ( $\chi^{2}$ [9] = 52.956, *p* = 0.000) hence the degrees of freedom were corrected with Greenhouse–Geisser estimates (ε = 0.368). All parameters for the ANOVA are available in Supplementary Table S10.

**Figure 12:**
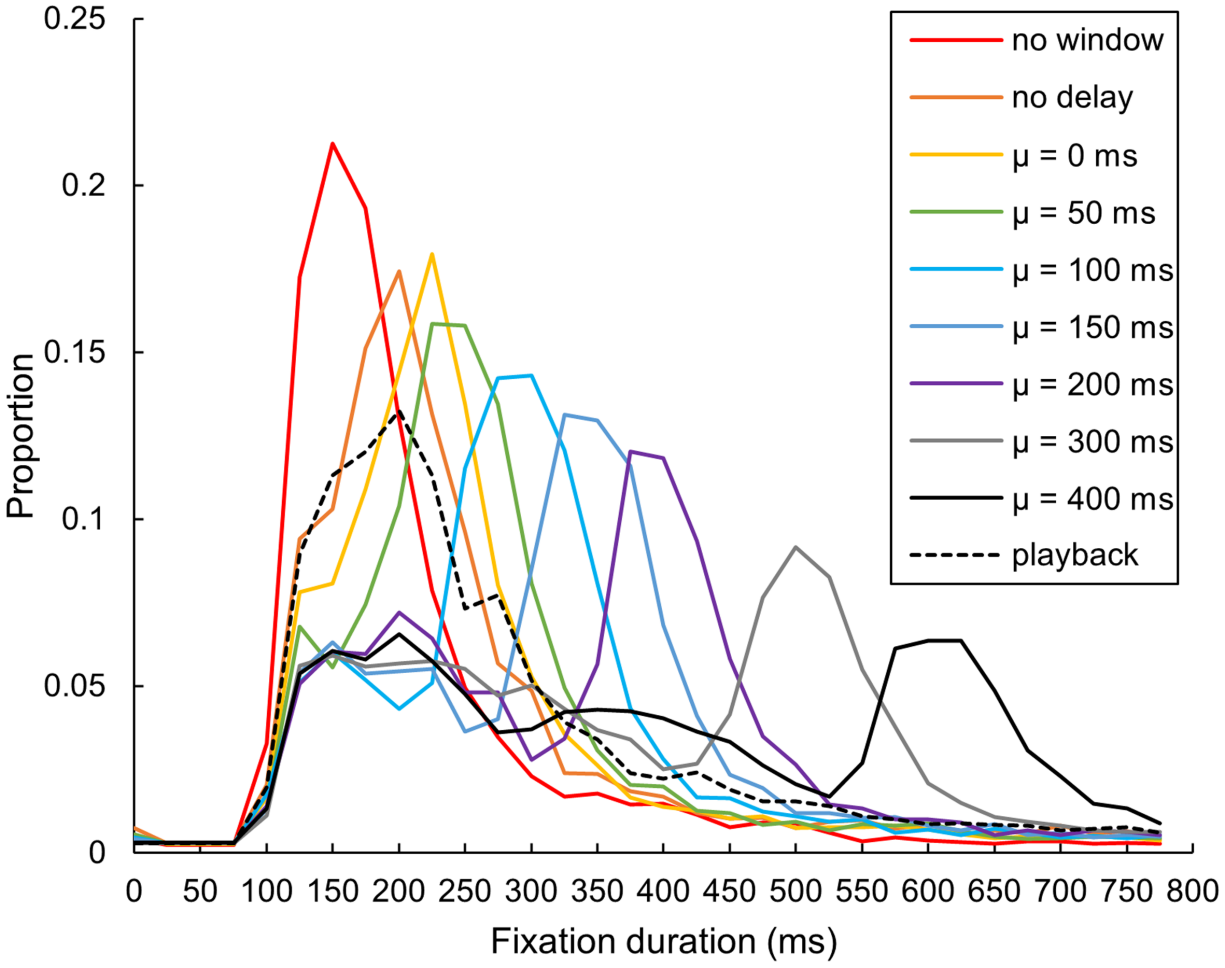
Relative distribution of fixation duration. Each participant’s fixation duration falling within every 25 ms bin, within a range of 0–800 ms, was calculated. The graph displays the means among participants, with a representative value of each bin placed at its minimum (e.g., the value of a 200–225 ms bin is shown at 200 ms on the horizontal axis) (*N* = 11). Note that the two participants did not conduct the “no window” condition.

The mean relative distribution of fixation duration was calculated for the 11 participants (Figure 12). In the no-window condition, the distribution mode appeared at 150 ms. Considering that the no-delay and playback conditions had similar modes at 200 ms, this seems to be due to the gaze-contingent window. As the delay was introduced, the mode shifted to 225 ms. Interestingly, from when the 𝜇 was 50 ms, we observed dual modes, one remaining around 125–225 ms, and another mode located at the position of the sum of 𝜇 and 200 ms (e.g., when the 𝜇 was 300 ms, the latter mode located at 500 ms).

**Figure 13:**
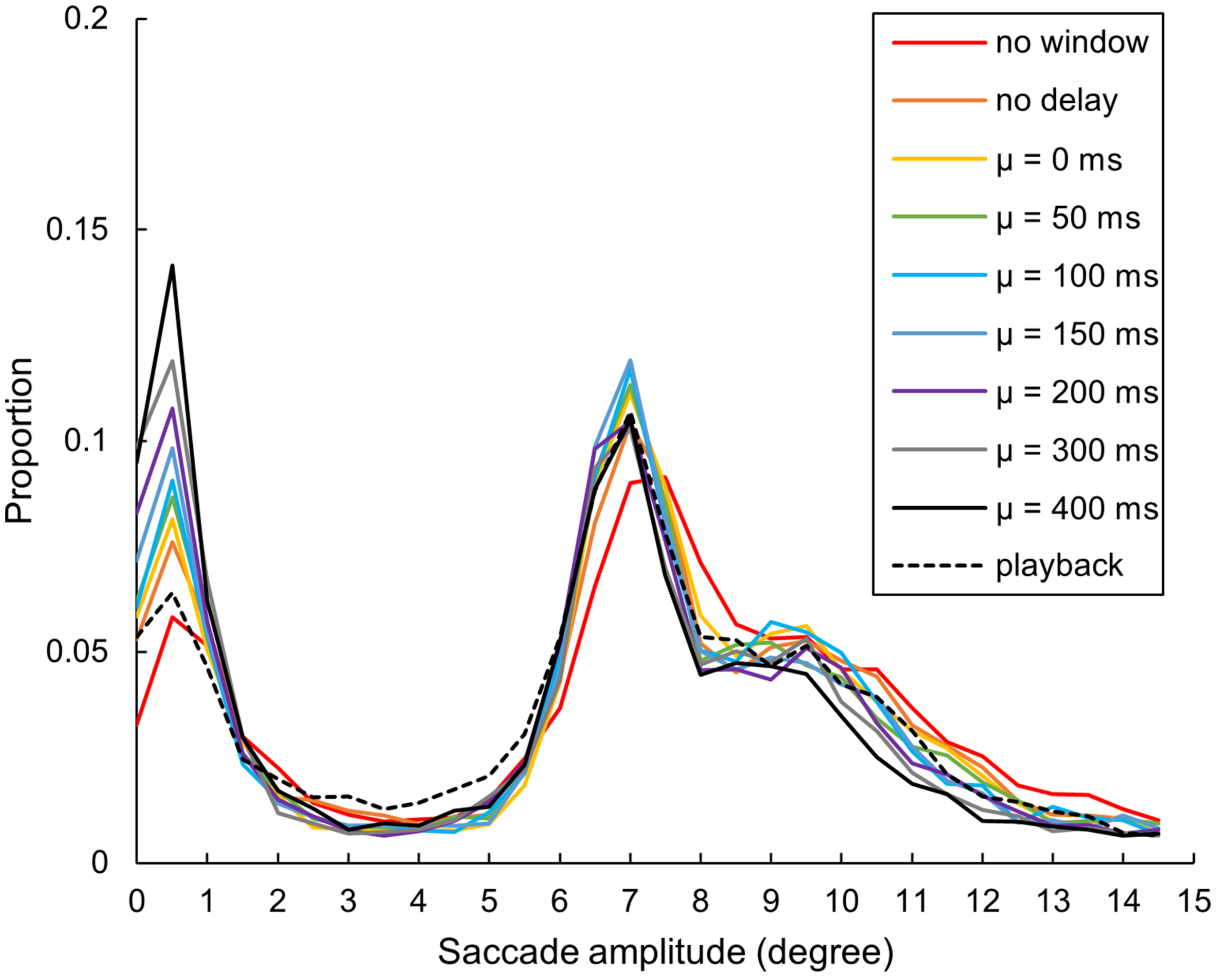
Relative distribution of saccade amplitude. Each participant’s saccade amplitude within every 0.5° bin, within a range of 0–15°, was calculated. The graph displays the means among participants, with a representative value of each bin placed at its minimum (e.g., the value of a 10–10.5° bin is shown at 10° on the horizontal axis) (*N* = 11). Note that the two participants did not conduct the “no window” condition.

The mean relative distribution of saccade amplitudes was again calculated among the 11 participants (Figure 13). Across all conditions, two distinct modes were observed: one at 1° and the other at 7°. Given that the shortest distance between the two characters was 8°, this likely explains the peak at 7°. Relative frequency below 1° seemed to rise with the gaze-contingent window when we compared no delay or playback to no window condition and further increased with the 𝜇.

## 4 Discussion

This study aimed to explore how a jittery delay influences the sense of agency and behaviors and to identify the statistical attributes that most effectively clarify the relationship between the sense of agency and eye movements under conditions of jittery delay.

### 4.1 Influences on the sense of agency

Categorical authorship reports showed that the self responses decreased as the delay increased. The dissociation between “delay” response frequency and response time in playback conditions and the predominance of other responses in the conditions suggests that the “delay” responses are more closely associated with the subjective sense of agency than with task difficulty. Likewise, the consistent authorship rating between one and two for playback conditions suggests that these ratings likely indicate participants’ perception of the authoring agent rather than task difficulty. Predominantly reported delay response (i.e., partial sense of agency) led to a more pronounced decrease in the proportion of the self-response than the decrease in the authorship rating where the rating of one implied total absence of the sense of agency. The decrease in authorship ratings with temporal delay aligned well with prior research that utilized hand movements as the primary action (Sato and Yasuda, 2005; Ebert and Wegner, 2010; Wen et al., 2015a,b; Shibuya et al., 2018). Specifically, our categorical authorship reports mirror those of Farrer et al. (2008) in two key aspects: (1) self responses decreased and delay responses increased alongside the temporal discrepancy, and (2) other responses were barely reported even at longer temporal discrepancies.

However, Farrer et al.’s (2013) study found participants misattributing authorship and selecting other responses with longer temporal discrepancies. These misattributions can be accounted for by the degrees of freedom in the action–feedback experimental paradigm. Both the current study and Farrer et al.’s (2008) study allowed participants to incorporate movement direction into their authorship judgments (gaze direction and joystick manipulation respectively). Farrer et al.’s (2013) study offered only a single degree of freedom (a single-button press), making it challenging for participants to claim authorship at extended temporal discrepancies. Such variations in sensory data across studies might further explain the disparities in the attenuation degree of subjective reports. For example, Farrer et al.’s (2013) study documented that an increase in the *partial control* response proportion began at longer temporal discrepancies than in our study. Wen (2019) suggested that copious sensory data would constrict the time window for a sense of agency. These disparities do not notably hinder our assertion that our study generally replicates prior research, even with the use of eye movement as the action modality.

### 4.2 Influences on behaviors

The fixation counts per trial showed no influence from the 𝜇 value, while the fixation counts per second exhibited a consistent decrease. This suggests that the number of fixations required to complete the visual search remained stable across different delay conditions, despite the delays disrupting their visual search time efficiency. Such a suggestion is further supported by the observed increase in response times. The observed decrease in fixation counts per second can be detailed by examining the fixation duration distributions. These distributions reveal two distinct patterns: (1) a shifting mode (hereafter referred to as “higher mode”) and (2) a consistent mode between 125 and 225 ms (hereafter, “lower mode”). The location of the higher mode closely correlates with the delay, moving in tandem with the 𝜇 value. Subtracting the 𝜇 value from the location of each higher mode across all delay conditions shows that all mode locations converge at 200 ms, except when 𝜇 is 0 ms, where it converges at 225 ms. This correlation likely reflects participant behavior waiting for the gaze-contingent window to update at their fixation point before reading the character, termed “wait-and-read” for the higher mode and identified as typical reading behaviors for the lower mode.

The distributions of the saccade amplitudes showed an effect of delay as the increase in the relative frequency of the 0–1° saccades. While we maintain some reservations about our camera-based eye-tracking capability to accurately record microsaccades, we can extrapolate the root causes of our findings from previous research on microsaccades. Previous research has proposed microsaccades as a proxy for covert attention allocation (Hafed and Clark, 2002; Engbert and Kliegl, 2003). We postulate that the separation between eye movement and attention allocation is a fitting explanation for our delayed gaze-contingent display. When the window tracks eye movements with a delay, it causes a distance between the participant’s gaze point on the monitor and the window. Such distance may necessitate participants to direct their attention to the window as part of their temporal mismatch perceptions while their gaze remains on the desired character to perform a visual search. As the delay increases, causing the distance between the window and their eye movement to expand, their frequency of covertly attending to the window increases.

Even though the action-effect delay could not significantly hinder the participants’ visual search, it did utilize their attentional resources. Although the low error rates and negligible influence of the μ value on fixation count per trial indicate a trivial impact of the delay on search behaviors, the variation in saccade amplitudes in response to 𝜇 values suggests an adaptive attention distribution and needs further exploration. Decreased visual search efficiency suggests higher attentional demands on participants’ working memory. The adoption of two fixation strategies indicates that participants chose to “wait and read” to reduce these demands. Those eye movement results hint at a possible proportional relationship between the amount of attention consumed and the length of the delay. However, this relationship cannot be confirmed since the delay did not affect task performance. Although attention is required for action-effect comparison (Wen et al., 2016) and is a limited resource essential for experiencing agency (Hon, 2017), our study, showing sustained search performance and a steady decline in subjective authorship, indicates that visual search and the sense of agency adequately shared the available attentional load. These findings underscore the importance of further manipulations concerning task difficulty.

### 4.3 Statistical attributes of jitter on the sense of agency

Comparison with constant delay conditions (Kim and Yoshida, 2023) revealed no significant differences in either the subjective report parameters or the fixation counts per second. The relative distributions of fixation durations and saccade amplitudes also did not show any noticeable impacts. These results suggest that the minimum value of the distribution predominantly influences the sense of agency and eye movements. In generalizing our findings, qualitative differences in fitted values should be noted. Plots of fitted values per participant (Supplementary Figures S8, S9) indicate that jittery delay may result in less uncertainty and require shorter delays to impair the sense of agency. Further research is needed to validate our findings. Furthermore, these conclusions are constrained by our choice of distribution, specifically σ set at 50 ms.

Direct comparisons with previous studies are scarce due to methodological differences, such as continuous action–effect interactions and the application of jittery distributions across delay ranges. Normoyle et al. (2014) provide a partially relevant comparison by manipulating the θ value of a gamma distribution to achieve variances of 0, 50, 100, and 150 ms, with a constant mean of 200 ms. In their study, participants perceived delays only at the highest variance of 150 ms. Our study employed a truncated normal distribution with a variance of 628.29 ms across all delay conditions, identifying a subjective report threshold of 50% at a delay of 94 ms, the distribution’s minimum value, with a mean delay of 130.14 ms. While direct comparisons with gamma distributions are challenging, our findings suggest a broader variance in threshold delay (mean = 130 ms, variance = 628 ms, min = 94 ms, max = 194 ms) compared to previous studies (mean = 200 ms, variance = 150 ms). In contrast with our truncated distribution, previous studies likely encountered occasional longer delays (e.g., over 400 ms) due to low-probability extreme delays, which could explain the perceived temporal delay at a smaller variance. This discrepancy raises questions about whether perceptions of temporal mismatch are more influenced by delays near a distribution’s minimum rather than its maximum value, prompting the need for future research with a more extensive range of delays.

The above experiments suggest that the threshold for perceiving delays may depend on both the length of the delay and its frequency of occurrence. The sporadic extended delay is critical when considering data packet transmission over the internet; an extended delay in one packet often predicts similar delays in subsequent packets (Bolot, 1993). Furthermore, packet loss tends to occur in clusters, affecting consecutive packets (Jiang and Schulzrinne, 2000). In the realm of continuous action-effect HCI, even brief experiences of such sporadic, consecutive extended delays can significantly impair the sense of agency and potentially alter user behavior. Therefore, future research should not only encompass experiments with a wider range of delays but also investigate the effects of varying occurrence rates of the delays.

### 4.4 Gaze-contingent display

Low error rates and significant response time differences in target presence conditions confirm participants navigated the visual search in a serial manner without any skipping, experiencing the designed inconvenience of jittery delays. The influences of delays on subjective reports and behaviors validate our design approach and underscore the efficacy of jittery delays. Also, we observed no decrease in the overall saccade amplitude, consistently recording 6–8° saccades across all conditions. The 3 × 3 virtual grid utilized in our experiment was considered to prevent reductions in saccade amplitude caused by the gaze-contingent window, thereby distinguishing our methodology from those previous researches where saccade amplitudes are reported to decrease with peripheral blurring gaze-contingent window (Reingold et al., 2003; Foulsham et al., 2011; Laubrock et al., 2013; Cajar et al., 2016a,b). Hence, the eye-gaze interface that we implemented effectively minimized the effect of the window, allowing us to focus solely on investigating the effects of delay.

## 5 Conclusion

In conclusion, our investigation into the effects of jittery delays on subjective authorship and motor commands uncovers an intricate relationship between the perception of delay and the refinement of actions, providing a novel perspective on how delays influence user interaction with gaze-contingent interfaces. This study stands as the first systematic exploration of the influence of jittery delays on the sense of agency, marking a significant advancement in our understanding of eye-gaze human-computer interaction under network conditions characterized by variability in delay. Our findings illuminate the potential of eye-gaze HCI to detect alterations in gaze behavior as indicators of users’ delay perception, laying the groundwork for the development of delay-tolerant interfaces that can adapt to the inherent variability in network communications. The insights derived from this research contribute to the design principles for more resilient and efficient gaze-contingent interfaces, enhancing user experience in environments affected by jittery delays.

### 5.1 Limitations and further research

This study has certain limitations, such as a participant pool constrained to a specific age bracket and the use of a specific distribution. An explicit questionnaire may not fully capture the sense of agency (e.g., the feeling of agency proposed by Synofzik et al., 2008). In addition, subjective tools, such as rating scales and response criteria, have inherent limitations and are often prone to systematic bias. Utilizing intentional binding with the time interval estimates paradigm (Humphreys and Buehner, 2009), or the Libet clock paradigm (Haggard et al., 2002) is another option. However, in these cases, the chronostasis effect, in which perceived time is dilated immediately after a saccade, is problematic when designing an experiment (Yarrow et al., 2001). Furthermore, investigating jittery delay in a continuous action-effects system would be impossible with intentional binding paradigms. Future work could potentially benefit from objective measures using signal detection frameworks. Also, the altered eye movements in a temporally delayed gaze-contingent display may not be triggered by other types of stimuli (e.g., natural scenes).

While this study provides valuable insights into the impact of jittery delays on gaze-contingent HCI, it does not directly explore the origins of the sense of agency, particularly whether it stems from internal comparisons between intentional oculomotor signals and visual feedback or from comparisons within various contexts (e.g., within visual feedback or between goals and results). This question remains a theoretical limitation, extending beyond the scope of our objectives yet important for understanding the full spectrum of factors influencing the sense of agency in eye-gaze HCI. Establishing a baseline condition, such as using transcranial magnetic stimulation to induce unintentional actions (Haggard et al., 2002; Haggard and Clark, 2003), would be critical for addressing this aspect comprehensively, posing a challenge for future research. This limitation underscores the need for further investigation to delineate the mechanisms of delay perception and their implications for the design and evaluation of gaze-contingent interfaces.

## Data availability statement

The datasets presented in this study can be found in online repositories. The names of the repository/repositories and accession number(s) can be found in the article/Supplementary material.

## Ethics statement

The studies involving humans were approved by The Tokyo Institute of Technology Ethics Review Committee for Epidemiological Research. The studies were conducted in accordance with the local legislation and institutional requirements. The participants provided their written informed consent to participate in this study.

## Author contributions

JK: Writing – review & editing, Writing – original draft, Visualization, Validation, Software, Resources, Methodology, Investigation, Funding acquisition, Formal analysis, Data curation, Conceptualization. TY: Writing – review & editing, Visualization, Validation, Supervision, Resources, Project administration, Methodology, Funding acquisition, Formal analysis, Conceptualization.

## References

[ref1] Anvari M. Broderick T. Stein H. Chapman T. Ghodoussi M. Birch D. W.. (2005). The impact of latency on surgical precision and task completion during robotic-assisted remote telepresence surgery. Comput. Aided Surg. 10, 93–99. doi: 10.3109/10929080500228654, PMID: 16298920

[ref2] Blakemore S.-J. Wolpert D. M. Frith C. D. (1998). Central cancellation of self-produced tickle sensation. Nat. Neurosci. 1, 635–640. doi: 10.1038/2870, PMID: 10196573

[ref3] Blakemore S.-J. Wolpert D. Frith C. (2000). Why canʼt you tickle yourself? Neuroreport 11, R11–R16. doi: 10.1097/00001756-200008030-00002, PMID: 10943682

[ref4] Blakemore S.-J. Wolpert D. M. Frith C. D. (2002). Abnormalities in the awareness of action. Trends Cogn. Sci. 6, 237–242. doi: 10.1016/S1364-6613(02)01907-1 12039604

[ref5] Bolot J.-C. (1993). End-to-end packet delay and loss behavior in the internet. SIGCOMM Comput. Commun. Rev. 23, 289–298. doi: 10.1145/167954.166265

[ref6] Brandi M.-L. Kaifel D. Lahnakoski J. M. Schilbach L. (2020). A naturalistic paradigm simulating gaze-based social interactions for the investigation of social agency. Behav. Res. Methods 52, 1044–1055. doi: 10.3758/s13428-019-01299-x, PMID: 31712998 PMC7280341

[ref7] Bridgeman B. (2010). How the brain makes the world appear stable. Iperception 1, 69–72. doi: 10.1068/i0387, PMID: 23397002 PMC3563054

[ref8] Cajar A. Engbert R. Laubrock J. (2016a). Spatial frequency processing in the central and peripheral visual field during scene viewing. Vis. Res. 127, 186–197. doi: 10.1016/j.visres.2016.05.008, PMID: 27491705

[ref9] Cajar A. Schneeweiß P. Engbert R. Laubrock J. (2016b). Coupling of attention and saccades when viewing scenes with central and peripheral degradation. J. Vis. 16:8. doi: 10.1167/16.2.8, PMID: 27271524

[ref10] Cavanaugh J. Berman R. A. Joiner W. M. Wurtz R. H. (2016). Saccadic corollary discharge underlies stable visual perception. J. Neurosci. 36, 31–42. doi: 10.1523/JNEUROSCI.2054-15.2016, PMID: 26740647 PMC4701964

[ref11] Chen H. Liu Z. Huang P. Kuang Z. (2022). Time-delay modeling and simulation for relay communication-based space Telerobot system. IEEE Trans Syst Man Cybern Syst 52, 4211–4222. doi: 10.1109/TSMC.2021.3090806

[ref12] Claxton G. (1975). Why Can’t we tickle ourselves? Percept. Mot. Skills 41, 335–338. doi: 10.2466/pms.1975.41.1.335, PMID: 1178428

[ref13] Claypool M. Tanner J. (1999). “The effects of jitter on the peceptual quality of video” in Proceedings of the seventh ACM international conference on multimedia (part 2) (New York, NY: Association for Computing Machinery), 115–118.

[ref14] Ebert J. P. Wegner D. M. (2010). Time warp: authorship shapes the perceived timing of actions and events. Conscious. Cogn. 19, 481–489. doi: 10.1016/j.concog.2009.10.002, PMID: 19896868 PMC2836403

[ref15] Emery N. J. (2000). The eyes have it: the neuroethology, function and evolution of social gaze. Neurosci. Biobehav. Rev. 24, 581–604. doi: 10.1016/S0149-7634(00)00025-7, PMID: 10940436

[ref16] Engbert R. Kliegl R. (2003). Microsaccades uncover the orientation of covert attention. Vis. Res. 43, 1035–1045. doi: 10.1016/S0042-6989(03)00084-1, PMID: 12676246

[ref17] Farrer C. Bouchereau M. Jeannerod M. Franck N. (2008). Effect of distorted visual feedback on the sense of agency. Behav. Neurol. 19, 53–57. doi: 10.1155/2008/425267, PMID: 18413918 PMC5452467

[ref18] Farrer C. Valentin G. Hupé J. M. (2013). The time windows of the sense of agency. Conscious. Cogn. 22, 1431–1441. doi: 10.1016/j.concog.2013.09.010 24161792

[ref19] Foulsham T. Teszka R. Kingstone A. (2011). Saccade control in natural images is shaped by the information visible at fixation: evidence from asymmetric gaze-contingent windows. Atten. Percept. Psychophys. 73, 266–283. doi: 10.3758/s13414-010-0014-5, PMID: 21258925

[ref20] Frith C. D. Blakemore S.-J. Wolpert D. M. (2000). Explaining the symptoms of schizophrenia: abnormalities in the awareness of action. Brain Res. Rev. 31, 357–363. doi: 10.1016/S0165-0173(99)00052-1, PMID: 10719163

[ref21] Gallagher S. (2000). Philosophical conceptions of the self: implications for cognitive science. Trends Cogn. Sci. 4, 14–21. doi: 10.1016/S1364-6613(99)01417-5, PMID: 10637618

[ref22] García B. Gortázar F. Gallego M. Hines A. (2020). Assessment of QoE for video and audio in WebRTC applications using full-reference models. Electronics (Basel) 9:462. doi: 10.3390/electronics9030462

[ref23] Gettys J. Nichols K. (2012). Bufferbloat: dark buffers in the internet. Commun. ACM 55, 57–65. doi: 10.1145/2063176.2063196

[ref24] Gregori Grgič R. Crespi S. A. de’Sperati C. (2016). Assessing self-awareness through gaze agency. PLoS One 11:e0164682. doi: 10.1371/journal.pone.0164682, PMID: 27812138 PMC5094589

[ref25] Grynszpan O. Simonin J. Martin J.-C. Nadel J. (2012). Investigating social gaze as an action-perception online performance. Front. Hum. Neurosci. 6:94. doi: 10.3389/fnhum.2012.00094, PMID: 22529796 PMC3330759

[ref26] Gulliver S. R. Ghinea G. (2007). The perceptual and attentive impact of delay and jitter in multimedia delivery. IEEE Trans. Broadcast. 53, 449–458. doi: 10.1109/TBC.2007.896955

[ref27] Gutzeit J. Weller L. Muth F. Kürten J. Huestegge L. (2024). Eye did this! Sense of agency in eye movements. Acta Psychol. 243:104121. doi: 10.1016/j.actpsy.2023.104121, PMID: 38199168

[ref28] Hafed Z. M. Clark J. J. (2002). Microsaccades as an overt measure of covert attention shifts. Vis. Res. 42, 2533–2545. doi: 10.1016/S0042-6989(02)00263-8, PMID: 12445847

[ref29] Haggard P. (2017). Sense of agency in the human brain. Nat. Rev. Neurosci. 18, 196–207. doi: 10.1038/nrn.2017.14 28251993

[ref30] Haggard P. Chambon V. (2012). Sense of agency. Curr. Biol. 22, R390–R392. doi: 10.1016/j.cub.2012.02.040 22625851

[ref31] Haggard P. Clark S. (2003). Intentional action: conscious experience and neural prediction. In: Consciousness and cognition, (Academic Press Inc.) 12, 695–707. doi: 10.1016/S1053-8100(03)00052-7, PMID: 14656511

[ref32] Haggard P. Clark S. Kalogeras J. (2002). Voluntary action and conscious awareness. Nat. Neurosci. 5, 382–385. doi: 10.1038/nn827 11896397

[ref33] Hon N. (2017). Attention and the sense of agency: a review and some thoughts on the matter. Conscious. Cogn. 56, 30–36. doi: 10.1016/j.concog.2017.10.004, PMID: 29035750

[ref34] Hua J. Cui Y. Yang Y. Li H. (2013). “Analysis and prediction of jitter of internet one-way time-delay for teleoperation systems” in 2013 11th IEEE international conference on industrial informatics (INDIN), 612–617.

[ref35] Humphreys G. R. Buehner M. J. (2009). Magnitude estimation reveals temporal binding at super-second intervals. J. Exp. Psychol. Hum. Percept. Perform. 35, 1542–1549. doi: 10.1037/a0014492, PMID: 19803655

[ref36] Ieiri S. Hashizume M. (2011). Development of surgical assisted system for remote medicine. Med. Biol. Eng. 49, 673–677. doi: 10.11239/jsmbe.49.673

[ref37] Imaida T. Yokokohji Y. Doi T. Oda M. Yoshikawa T. (2004). Ground-space bilateral teleoperation of ETS-VII robot arm by direct bilateral coupling under 7-s time delay condition. IEEE Trans. Robot. Autom. 20, 499–511. doi: 10.1109/TRA.2004.825271

[ref38] Jiang W. Schulzrinne H. (2000). Modeling of packet loss and delay and their effect on real-time multimedia service quality, In: Proceedings of NOSSDAV, 1–10.

[ref39] Kim S. Lee J. Y. Sung D. (2003). Shifted gamma distribution model for long-range dependent internet traffic. Commun. Lett. IEEE 7, 124–126. doi: 10.1109/LCOMM.2002.808400

[ref40] Kim E. Peysakhovich V. Roy R. N. (2021). “Impact of communication delay and temporal sensitivity on perceived workload and teleoperation performance” in ACM symposium on applied perception 2021 (New York, NY: Association for Computing Machinery).

[ref41] Kim J. Yoshida T. (2023). Sense of agency at temporally delayed gaze-contingent display. Available at: psyarxiv.com/jd74n (Accessed March 3, 2024). 10.1371/journal.pone.0309998 PMC12139658 39241025

[ref42] Laubrock J. Cajar A. Engbert R. (2013). Control of fixation duration during scene viewing by interaction of foveal and peripheral processing. J. Vis. 13:11. doi: 10.1167/13.12.11, PMID: 24133291

[ref43] Microsoft (n.d.). Get started with eye control in windows. Retrieved from Microsoft support. Available at: https://support.microsoft.com/en-us/windows/get-started-with-eye-control-in-windows-1a170a20-1083-2452-8f42-17a7d4fe89a9 (Accessed September 7, 2023).

[ref44] Moore J. W. (2016). What is the sense of agency and why does it matter? Front. Psychol. 7:1272. doi: 10.3389/fpsyg.2016.01272, PMID: 27621713 PMC5002400

[ref45] Musicant O. Botzer A. Shoval S. (2023). Effects of simulated time delay on teleoperators’ performance in inter-urban conditions. Transp. Res. F Traffic Psychol. Behav. 92, 220–237. doi: 10.1016/j.trf.2022.11.007

[ref46] Normoyle A. Guerrero G. Jörg S. (2014). “Player perception of delays and jitter in character responsiveness” in Proceedings of the ACM symposium on applied perception (New York, NY: Association for Computing Machinery), 117–124.

[ref47] Oboe R. Fiorini P. (1997). Issues on internet-based teleoperation. IFAC Proc. Vol. 30, 591–597. doi: 10.1016/S1474-6670(17)44322-9

[ref48] Pfeiffer U. Schilbach L. Timmermans B. Jording M. Bente G. Vogeley K. (2012). Eyes on the mind: investigating the influence of gaze dynamics on the perception of others in real-time social interaction. Front. Psychol. 3:537. doi: 10.3389/fpsyg.2012.00537, PMID: 23227017 PMC3512550

[ref49] Recht S. Grynszpan O. (2019). The sense of social agency in gaze leading. J. Multimodal User Interfaces 13, 19–30. doi: 10.1007/s12193-018-0286-y

[ref50] Reingold E. M. Loschky L. C. McConkie G. W. Stampe D. M. (2003). Gaze-contingent Multiresolutional displays: an integrative review. Hum. Factors 45, 307–328. doi: 10.1518/hfes.45.2.307.27235, PMID: 14529201

[ref51] Roy A. Pachuau J. L. Saha A. K. (2021). An overview of queuing delay and various delay based algorithms in networks. Computing 103, 2361–2399. doi: 10.1007/s00607-021-00973-3

[ref52] Sasaki Y. (2022). Evaluation of operability in tele-operation control of a hydraulic cylinder by the packet compensation method. J. MMIJ 138, 149–159. doi: 10.2473/journalofmmij.138.149

[ref53] Sato A. (2008). Both motor prediction and conceptual congruency between preview and action-effect contribute to explicit judgment of agency. Cognition 110, 74–83. doi: 10.1016/j.cognition.2008.10.011, PMID: 19038380

[ref54] Sato A. Yasuda A. (2005). Illusion of sense of self-agency: discrepancy between the predicted and actual sensory consequences of actions modulates the sense of self-agency, but not the sense of self-ownership. Cognition 94, 241–255. doi: 10.1016/j.cognition.2004.04.003, PMID: 15617673

[ref55] Scholcover F. Gillan D. J. (2023). Temporal sensitivity and latency during teleoperation: using track clearance to understand errors in future projection. Hum. Factors 65, 260–274. doi: 10.1177/00187208211011842, PMID: 33887982

[ref56] Sharma R. Pavlovic V. I. Huang T. S. (1998). Toward multimodal human-computer interface. Proc. IEEE 86, 853–869. doi: 10.1109/5.664275

[ref57] Sheridan T. B. (1993). Space teleoperation through time delay: review and prognosis. IEEE Trans. Robot. Autom. 9, 592–606. doi: 10.1109/70.258052

[ref58] Shibuya S. Unenaka S. Ohki Y. (2018). The relationship between the virtual hand illusion and motor performance. Front. Psychol. 9:2242. doi: 10.3389/fpsyg.2018.02242, PMID: 30515118 PMC6255939

[ref59] Shimada S. Qi Y. Hiraki K. (2010). Detection of visual feedback delay in active and passive self-body movements. Exp. Brain Res. 201, 359–364. doi: 10.1007/s00221-009-2028-6, PMID: 19830411

[ref60] Sperry R. W. (1950). Neural basis of the spontaneous optokinetic response produced by visual inversion. J. Comp. Physiol. Psychol. 43, 482–489. doi: 10.1037/h0055479, PMID: 14794830

[ref61] Sukhov A. M. Astrakhantseva M. A. Pervitsky A. K. Boldyrev S. S. Bukatov A. A. (2016). Generating a function for network delay. J. High Speed Netw. 22, 321–333. doi: 10.3233/JHS-160552

[ref62] Synofzik M. Vosgerau G. Newen A. (2008). Beyond the comparator model: a multifactorial two-step account of agency. Conscious. Cogn. 17, 219–239. doi: 10.1016/j.concog.2007.03.010, PMID: 17482480

[ref63] Timman S. Landgraf M. Haskamp C. Lizy-Destrez S. Dehais F. (2023). Effect of time-delay on lunar sampling tele-operations: evidences from cardiac, ocular and behavioral measures. Appl. Ergon. 107:103910. doi: 10.1016/j.apergo.2022.103910, PMID: 36334579

[ref64] Tobii (n.d.). Tobii Dynavox. Available at: https://www.tobiidynavox.com/ (Accessed September 7, 2023).

[ref65] von Holst E. (1954). Relations between the central nervous system and the peripheral organs. Br. J. Anim. Behav. 2, 89–94. doi: 10.1016/S0950-5601(54)80044-X

[ref66] Weiskrantz L. Elliott J. Darlington C. (1971). Preliminary observations on tickling oneself. Nature 230, 598–599. doi: 10.1038/230598a0, PMID: 4928671

[ref67] Wen W. (2019). Does delay in feedback diminish sense of agency? A review. Conscious. Cogn. 73:102759. doi: 10.1016/j.concog.2019.05.007, PMID: 31173998

[ref68] Wen W. Yamashita A. Asama H. (2015a). The influence of action-outcome delay and arousal on sense of agency and the intentional binding effect. Conscious. Cogn. 36, 87–95. doi: 10.1016/j.concog.2015.06.004, PMID: 26100602

[ref69] Wen W. Yamashita A. Asama H. (2015b). The sense of agency during continuous action: performance is more important than action-feedback association. PLoS One 10:e0125226. doi: 10.1371/journal.pone.0125226, PMID: 25893992 PMC4404253

[ref70] Wen W. Yamashita A. Asama H. (2016). Divided attention and processes underlying sense of agency. Front. Psychol. 7:35. doi: 10.3389/fpsyg.2016.00035, PMID: 26858680 PMC4729891

[ref71] Wolpert D. M. Flanagan J. R. (2001). Motor prediction. Curr. Biol. 11, R729–R732. doi: 10.1016/S0960-9822(01)00432-8 11566114

[ref72] Yarrow K. Haggard P. Heal R. Brown P. Rothwell J. C. (2001). Illusory perceptions of space and time preserve cross-saccadic perceptual continuity. Nature 414, 302–305. doi: 10.1038/35104551, PMID: 11713528

[ref73] Yokokohji Y. Imaida T. Yoshikawa T. (1999). Bilateral teleoperation under time-varying communication delay, In Proceedings 1999 IEEE/RSJ international conference on intelligent robots and systems. Human and environment friendly robots with high intelligence and emotional quotients (cat. No.99CH36289), Vol. 3, 1854–1859.

[ref74] Zhang W. He J. (2007). Modeling end-to-end delay using Pareto distribution. In: Second international conference on internet monitoring and protection (ICIMP 2007), 21.

