# Supplementary material for: Sense of agency at a gaze-contingent display with jittery temporal delay
Source: Front Psychol. 2024 May 17;15:1364076. doi: 10.3389/fpsyg.2024.1364076 (PMC11141391; doi:10.3389/fpsyg.2024.1364076)
Supplement: Supplementary file 1 [file Data_Sheet_1.docx]

Supplementary Material

The data and code for the current experiments are available via Open Science Framework at https://osf.io/x24pb/?view_only=585d2311a4704c029a4b1c4a9856339a

We examined whether participants strategically positioned their gaze between two neighboring characters to view both simultaneously, potentially reducing response times and affecting eye movements.

We set the minimum thresholds for eye movements to 0.1°, 30.0°/s, and 8000.0°/s^2^ to identify saccades. Any fixations that occurred outside the stimuli, began immediately before or after a blink or lasted less than 120 ms were excluded from our analyses.

We divided the stimuli into three areas of interest: 1) the gap region, where partial sections of two Chinese characters appeared within the gaze-contingent window when participants fixated (Supplementary Figure 1, black); 2) the word region, where only one character was visible within the window (Supplementary Figure 1, green); and 3) the null region, where no character was visible within the window (Supplementary Figure 1, gray). The mean fixation count per trial in each area (Supplementary Table 1) was calculated for each participant (270 trials). Very few fixations were observed in the gap region. This suggested that the participants did not intentionally gaze at two adjacent characters.

Supplementary Table 1. The count of fixations per trial for each region. The means and standard deviations among the participants were calculated. The data showed few fixations on regions apart from the word region, suggesting that behaviors unrelated to the task barely occurred.

| Regions | Mean | Standard deviation |
| --- | --- | --- |
| Gap region | 0.12 | 0.06 |
| Word region | 14.89 | 4.55 |
| Null region | 0.00 | 0.00 |


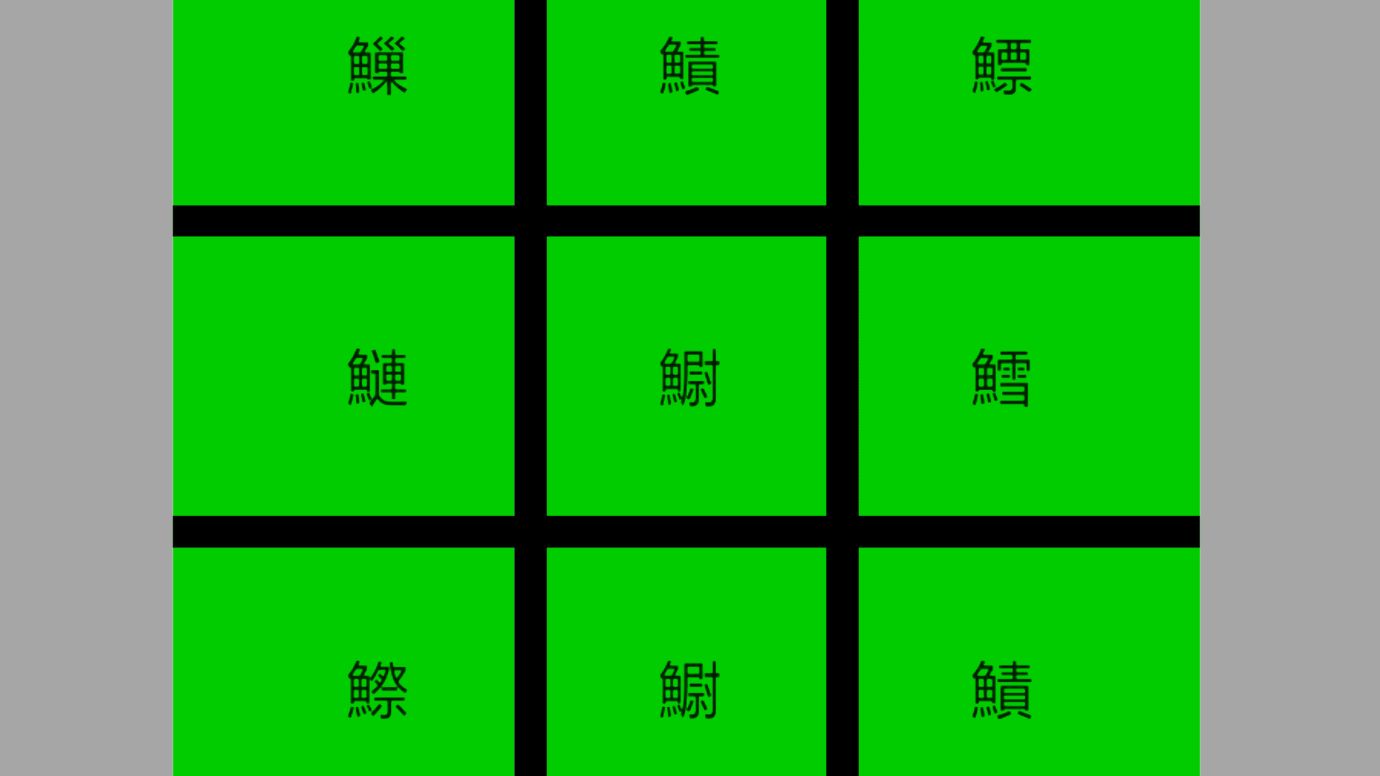


2 deg.

9 deg.

11 deg.

11 deg.

Supplementary Figure 1. Regions of interest on the stimulus. The stimuli were split into three distinct interest areas: 1) gap region, which reveals portions of two Chinese characters within the gaze-contingent window when the gaze was located (marked in black); 2) word region, rendering just one character within the window (highlighted in green); and 3) null region, which was unable to render any character within the window (denoted in grey).

The error rate per participant, condition, and the presence of the target were documented. The playback condition frequently exhibits the highest error rate. Participants struggled to inspect all eight surrounding characters in the playback condition because prerecorded gaze behavior often failed to reveal the entire character array.

Supplementary Table 2. The task error rate for each participant, each condition, and target existence.

|  | | Delayed window condition | | | | Playback condition | | | | No window condition | | | |  |
| --- | --- | --- | --- | --- | --- | --- | --- | --- | --- | --- | --- | --- | --- | --- |
| Participant | Stimuli | Trial counts | Error  trial counts | Error rate | Trial counts | | Error  trial counts | Error rate | Trial counts | | Error  trial counts | Error rate |  |  |
| 1 | no-target | 120 | 2 | 1.67% | 14 | | 0 | 0.00% |  | | | | | |
|  | target | 117 | 3 | 2.56% | 16 | | 6 | 37.50% |  |  |  |  |  |  |
|  | Total | 237 | 5 | 2.11% | 30 | | 6 | 20.00% |  |  |  |  |  |  |
| 2 | no-target | 120 | 0 | 0.00% | 16 | | 0 | 0.00% | 15 | | 0 | 0.00% |  |  |
|  | target | 119 | 4 | 3.36% | 14 | | 1 | 7.14% | 15 | | 0 | 0.00% |  |  |
|  | Total | 239 | 4 | 1.67% | 30 | | 1 | 3.33% | 30 | | 0 | 0.00% |  |  |
| 3 | no-target | 126 | 0 | 0.00% | 12 | | 0 | 0.00% | 15 | | 0 | 0.00% |  |  |
|  | target | 114 | 3 | 2.63% | 18 | | 6 | 33.33% | 15 | | 0 | 0.00% |  |  |
|  | Total | 240 | 3 | 1.25% | 30 | | 6 | 20.00% | 30 | | 0 | 0.00% |  |  |
| 4 | no-target | 120 | 0 | 0.00% | 17 | | 0 | 0.00% | 15 | | 0 | 0.00% |  |  |
|  | target | 120 | 0 | 0.00% | 13 | | 2 | 15.38% | 15 | | 0 | 0.00% |  |  |
|  | Total | 240 | 0 | 0.00% | 30 | | 2 | 6.67% | 30 | | 0 | 0.00% |  |  |
| 5 | no-target | 123 | 1 | 0.81% | 14 | | 0 | 0.00% | 15 | | 0 | 0.00% |  |  |
|  | target | 117 | 0 | 0.00% | 16 | | 1 | 6.25% | 15 | | 0 | 0.00% |  |  |
|  | Total | 240 | 1 | 0.42% | 30 | | 1 | 3.33% | 30 | | 0 | 0.00% |  |  |
| 6 | no-target | 119 | 0 | 0.00% | 18 | | 0 | 0.00% | 15 | | 0 | 0.00% |  |  |
|  | target | 121 | 5 | 4.13% | 12 | | 4 | 33.33% | 15 | | 0 | 0.00% |  |  |
|  | Total | 240 | 5 | 2.08% | 30 | | 4 | 13.33% | 30 | | 0 | 0.00% |  |  |
| 7 | no-target | 124 | 2 | 1.61% | 13 | | 0 | 0.00% | 15 | | 0 | 0.00% |  |  |
|  | target | 116 | 2 | 1.72% | 17 | | 8 | 47.06% | 15 | | 0 | 0.00% |  |  |
|  | Total | 240 | 4 | 1.67% | 30 | | 8 | 26.67% | 30 | | 0 | 0.00% |  |  |
| 8 | no-target | 119 | 0 | 0.00% | 18 | | 0 | 0.00% | 16 | | 0 | 0.00% |  |  |
|  | target | 121 | 0 | 0.00% | 12 | | 2 | 16.67% | 14 | | 0 | 0.00% |  |  |
|  | Total | 240 | 0 | 0.00% | 30 | | 2 | 6.67% | 30 | | 0 | 0.00% |  |  |
| 9 | no-target | 121 | 0 | 0.00% | 16 | | 0 | 0.00% | 15 | | 0 | 0.00% |  |  |
|  | target | 119 | 3 | 2.52% | 14 | | 2 | 14.29% | 15 | | 0 | 0.00% |  |  |
|  | Total | 240 | 3 | 1.25% | 30 | | 2 | 6.67% | 30 | | 0 | 0.00% |  |  |
| 10 | no-target | 122 | 0 | 0.00% | 15 | | 0 | 0.00% |  | | | | | |
|  | target | 118 | 3 | 2.54% | 15 | | 3 | 20.00% |  |  |  |  |  |  |
|  | Total | 240 | 3 | 1.25% | 30 | | 3 | 10.00% |  |  |  |  |  |  |
| 11 | no-target | 125 | 1 | 0.80% | 12 | | 0 | 0.00% | 15 | | 0 | 0.00% |  |  |
|  | target | 115 | 2 | 1.74% | 18 | | 5 | 27.78% | 15 | | 0 | 0.00% |  |  |
|  | Total | 240 | 3 | 1.25% | 30 | | 5 | 16.67% | 30 | | 0 | 0.00% |  |  |

#

Supplementary Table 3. The list of Chinese characters utilized in the stimuli. Each image encompassed nine characters, each featuring the same number of strokes.

| Number of strokes | Chinese Characters |
| --- | --- |
| 15 | 魴 魪 䰻 魥 魭 魧 魦 魣 魳 魫 魶 魹 魷 魨 魸 䰽 魬 魮 魵 䰷 魲 |
| 16 | 鮎 鮒 鮃 鮓 鮑 鮗 鮖 䱀 魼 鮔 䱁 鮏 鮀 鮐 鮊 鮁 鮍 魾 鮇 鮅 鮄 鮋 䱂 魿 䱈 鮌 鮉 䱇 |
| 17 | 鮮 鮪 鮭 鮫 鮟 鮨 鮠 鮴 鮣 鮱 鮰 鮚 鮬 鮯 䱎 䱍 鮥 鮞 鮛 鮡 鮧 鮦 鮩 鮲 |
| 18 | 鯉 鯀 䱔 鯒 鯑 鯏 鮹 鯆 鯎 鯇 鯁 鯋 䱒 鮻 䱌 鮼 鯐 鮾 鮵 鮿 鮷 䱐 鮸 |
| 19 | 鯨 鯖 鯵 鯛 鯣 鯢 鯤 鯲 鯡 鯔 鯱 鯰 鯘 䱤 鯕 䱡 䱟 鯝 䱜 鯞 䱙 鯧 鯩 鯜 鯳 鯯 鰙 鯫 鯮 鯟 鯥 鯪 |
| 20 | 鰐 鰍 鰓 鰉 鰔 鰕 鰌 鰈 鰆 鰒 鰊 鰄 鰛 鯹 䱭 鰀 鰋 鰅 鯸 鯶 鰇 鯺 鯽 鯼 鰂 鰖 鯷 䱱 鰚 鰏 鯾 鯿 鰘 鰑 鰦 |
| 21 | 鰯 鰭 鰮 鰥 鰤 鰰 鰡 䱽 鰞 鰪 䱵 䱻 鰜 鰬 鰝 䱹 鰣 鰠 鰧 鰨 鰢 鰟 鰩 鰫 |
| 22 | 鱈 鰺 鰻 鱆 鱇 鰾 鰱 䲁 鰳 䲅 鰶 鰼 鰽 鱃 鱂 鰷 鰿 鱄 䲃 鱁 鱅 |
| 23 | 鰹 鱒 鱚 鱉 鱊 鱛 鱎 鱖 鱑 鱏 鱘 鱓 鱔 鱍 鱕 鱝 |
| 24 | 鱗 鱠 鱧 鱫 鱜 鱞 鱤 鱥 鱪 鱐 鱢 鱣 鱩 鱯 鱮 |

We provide a concise overview of a previous study (Kim & Yoshida, 2023) in which constant delays, ranging from 0 to 4000 ms, were implemented. In this study, we retrieved only the results with a low-visibility window.

## Participants

Eight participants (M = 24.4; SD = 1.5; range: 22–27; two undergraduate and six master’s students; five Japanese, two Koreans, and one Chinese) provided informed consent, and their data were analyzed.

## Stimuli

Two types of blurred images were used to establish two levels of window visibility: low visibility and high visibility (Supplementary Figure 2). This was set to further investigate the potential influence of visibility on the sense of agency.


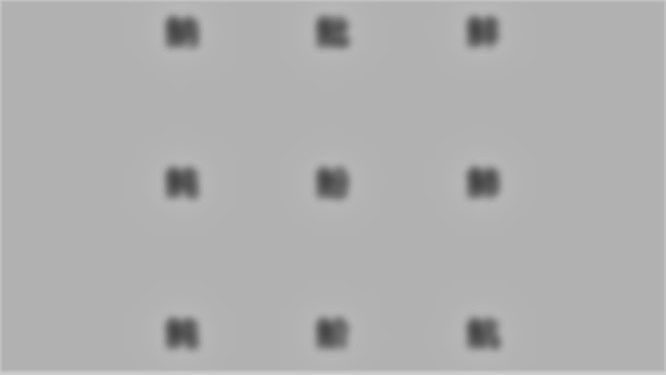

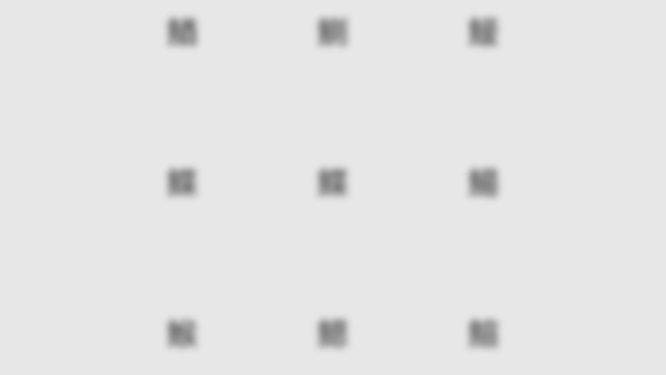

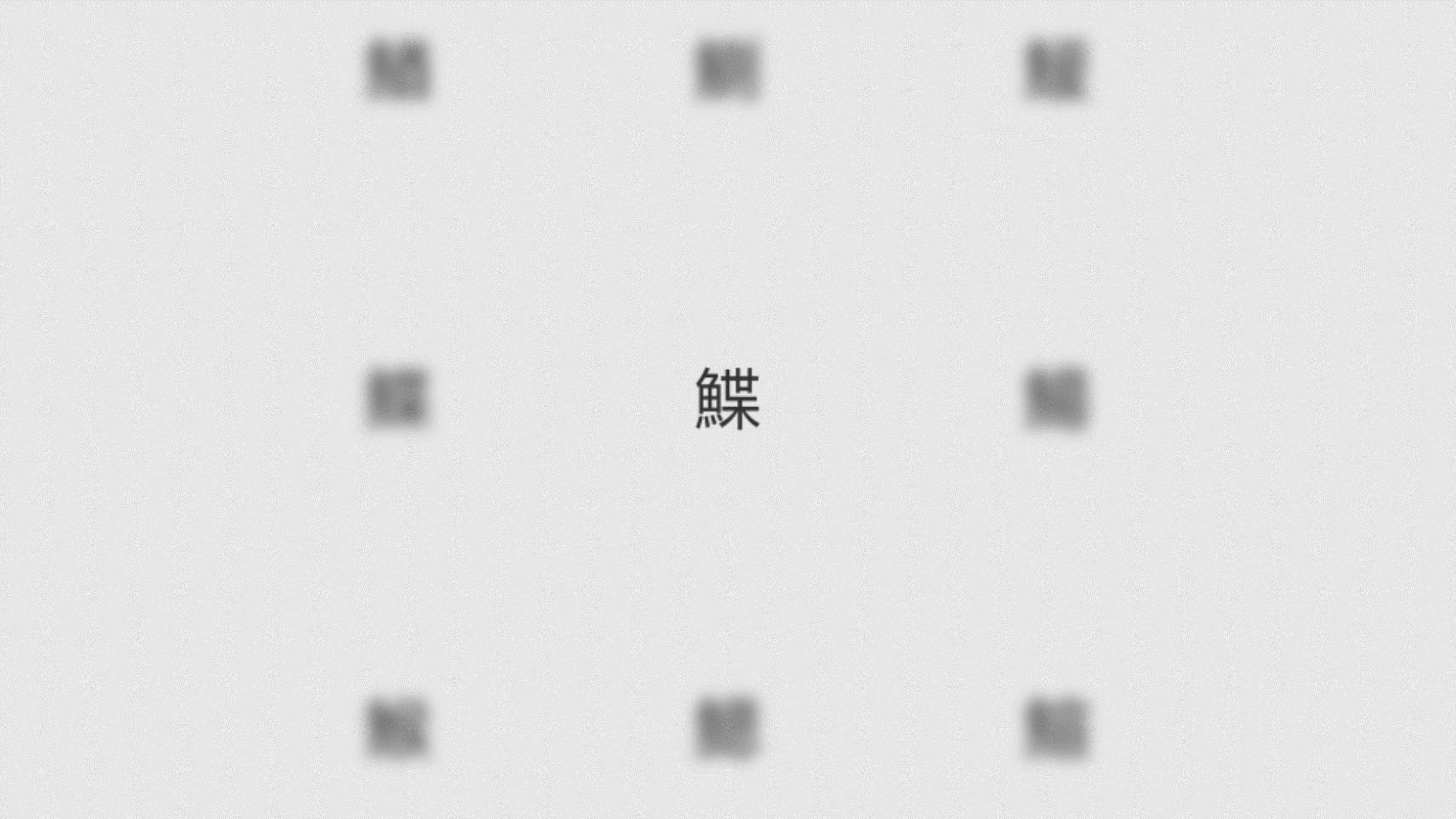

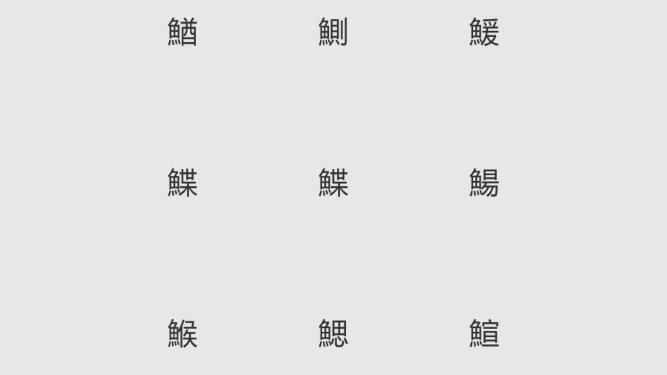

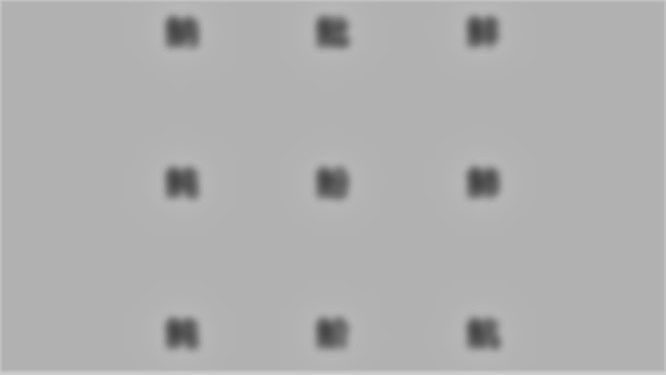

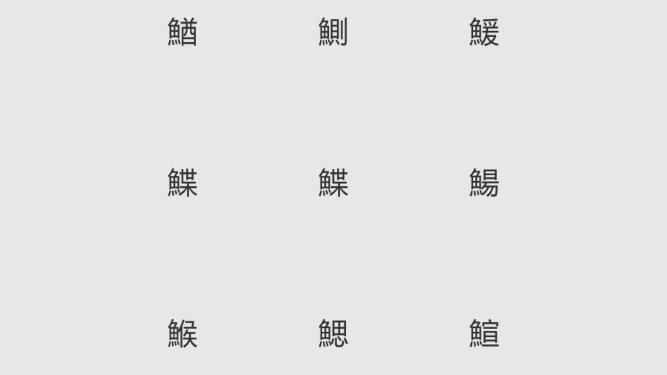


Supplementary Figure 2. Stimulus design. Representation of the stimulus. A, top-left) blurred image used for the low-visibility window. B, top-right) Blurred and darkened images are used for the high-visibility window. C, bottom-left) An example of a stimulus with a low-visibility window is when a participant fixated on the central Character. D, bottom-right) An example of the stimulus with the high-visibility window.

## Method

Apparatuses and procedures identical to those used in the current study were employed, with the primary difference being the introduction of a constant temporal delay. Each session consisted of 18 trials, which included 15 levels of delay (0, 30, 60, 100, 200, 300, 400, 500, 1,000, 1,500, 2,000, 2,500, 3,000, 3,500, and 4,000 ms), along with a playback trial, all of which were presented in a randomized sequence following two practice trials. The participants completed 30 sessions for each window visibility level, with 60 sessions per participant. Additionally, the participants completed 30 no-window trials, in which they performed the task without the visual constraint of a gaze-contingent window.

## Data and Code Availability

The data and code for the experiments of the previous study (Kim & Yoshida, 2023) are available via Open Science Framework at https://osf.io/x5w3d/?view_only=13a59dd61add49dfa0f2dabc90f75903

## Results

The average response time and standard deviations were calculated based on each participant's mean response time under different conditions, including the presence or absence of the target and level of window visibility (Supplementary Figure 3). A visibility × target × delay repeated measures ANOVA with Greenhouse–Geisser correction was performed on the participants’ average response times under the delayed window condition. A significant main effect of delay (*F* [1.350, 9.450] = 53.906, *p* = 0.000) verified that the response time increased with increased delay. The main effect of target presence also emerged as significant (*F* [1, 7] = 18.180, *p* = 0.004), with longer response times noted for stimuli without a target (*M* = 9586 ms) and faster response times for target-containing stimuli (*M* = 7693 ms). The mean difference of 1894 ms was significant in the subsequent pairwise comparison with Bonferroni correction (p = 0.004). However, visibility was not significant (*F* [1, 7] = .007, *p* = 0.936). The delay × target interaction was significant (*F* [3.532, 24.723] = 4.265, *p* = 0.011).

Supplementary Figure 3. Means and standard deviations of the response times.

The questionnaire investigated the categorical authorship variable, which included three levels: self, delay, and others. We calculated the average and standard deviation of the participants for each condition and window visibility with respect to the proportion of each response, as illustrated in Supplementary Figure 4. The results are depicted using a linear x-axis (Supplementary Figure 4A) and a nonlinear x-axis (Supplementary Figure 4B).

In the condition with a delayed window, excluding the playback condition, we used two generalized linear mixed models (GLMM; SPSS 24.0). This analysis compared *self* responses to *delay* responses, and *other* responses to *delay* responses, utilizing a multinomial probability distribution and generalized logit link function. For the random effects, we selected participants with variance components as the covariance structure. Window visibility and temporal delay were treated as fixed effects. Due to data loss in the subjective report, we excluded 42 responses, leaving 7,158 responses for analysis. Because the sample size was small and the data were unbalanced due to partial data loss, the Satterthwaite Formula was employed to approximate the degrees of freedom.

A significant main effect of delay was observed (*b* = -5.770, *SE* = 1.846, *t* = -3.126, *p* = .002) when comparing self to delay responses. With an increase in delay, self-responses were chosen less often than delay responses. Comparing low-visibility windows to high-visibility ones (LV – HV), window visibility had a significant main effect (*b* = 1.175, *SE* = 0.378, *t* = 3.111, *p* = .002). This suggests that the self- response was most frequent in low-visibility windows. As depicted in Supplementary Figure 4, the proportion of self responses from participants declined with an increase in delay, particularly in the low-visibility window compared to the high-visibility window. This suggests that participants could more easily discern the temporal incongruency between efferents and afferents signals with a high-visibility window. This pattern was further supported by the significant interaction of delay × LV – HV (*b* = -6.223, *SE* = 1.965, *t* = -3.168, *p* = .002).

In the comparison of other responses to delay responses, the main effect of delay was not significant (*b* = -0.068, *SE* = 0.213, *t* = -0.318, *p* = .750), indicating that other responses were seldom chosen in the delay condition. When comparing low-visibility windows to high-visibility ones, window visibility was not significant as a main effect (*b* = -0.088, *SE* = 0.296, *t* = -0.296, *p* = .767), suggesting that the other response was scarcely selected in both window conditions. There was also no significant interaction between delay and LS-MS (*b* = -0.080, *SE* = 0.248, *t* = -0.322, *p* = .747).

Supplementary Figure 4. The proportion of participants reported authorship (self, delay, or other). Means and standard deviations were computed among the participants. The findings are depicted with both a linear x-axis (A, top) and a nonlinear x-axis (B, bottom).

The degree of authorship was measured using a 6-point rating scale, where one represented “I could not manipulate the window at all,” and six signified “I completely manipulated the window.” We calculated the mean and standard deviation of these ratings among participants for every condition, considering window visibility (Supplementary Figure 5). We conducted a repeated-measures ANOVA with Greenhouse–Geisser correction, considering visibility and delay, on the condition with the delayed window. The effect of the delay was highly significant (*F* [2.199, 15.391] = 149.930, *p* = 0.000). However, the effects of visibility (*F* [1, 7] = .118, *p* = 0.741) and the interaction between visibility and delay (*F* [2.874, 20.120] = .518, *p* = 0.667) were not significant.

Supplementary Figure 5. Authorship ratings for each salient condition. The degree of authorship was measured using a 6-point scale, with one representing “I could not manipulate the window at all” and six representing “I completely manipulated the window.” The average authorship ratings were presented per window visibility and delay condition. The mean values and standard deviations were calculated among the participants.

We calculated the mean relative fixation duration frequency from the representative frequencies of the eight participants (Supplementary Figure 6). For each participant, the fixation duration frequency within each 25 ms bin in the 0-800 ms range was converted into a relative frequency for each condition, as the count of eye movements significantly varied between participants due to differing response times. Without a gaze-contingent window, the distribution mode was located roughly between 125 and 175 ms. With the introduction of a gaze-contingent window, the mode shifted to 175-225 ms for the 0 ms and 30 ms delay conditions and skewed negatively as the delay increased. Interestingly, from 200 ms to 500 ms delay, dual modes emerged: one located approximately at 175-225 ms and another that shifted with increasing delay. From a 1000 ms to 4000 ms delay, including the playback condition, the mode consistently occurred at 150-225 ms. No significant difference was noted between the two window visibility conditions.

We also calculated the mean relative saccade amplitude frequencies from the representative frequencies of the eight participants (Supplementary Figure 7). For each participant, the saccade amplitude frequency within each 0.5° bin in the 0-15° range was converted into a relative frequency for each condition to account for individual saccade count differences. Two distributions were generally observed across all conditions: one mode was located below 1° and another at 7-8°. The smallest distance between the two characters was 8°, likely causing a peak at 7-8°. The relative frequency below 1.5° seemed to increase with the gaze-contingent window compared to the no-window condition, and it further increased as the delay increased. This increasing trend of the relative frequency below 1.5° was not consistent from a delay of 1000 to 4000 ms in the high visibility condition. Again, no apparent difference was observed between the two window visibility conditions.

Supplementary Figure 6. Relative distribution of fixation duration. Each participant's fixation duration falling within every 25 ms bin, within a range of 0–800 ms, was calculated. The graph displays the means among participants, with a representative value of each bin placed at its minimum (e.g., the value of a 200–225 ms bin is shown at 200 ms on the horizontal axis). (top left [A] and right [B]) distributions with a low-visibility window. (bottom left [C] and right [D]) distributions with a high-visibility window.

Supplementary Figure 7. Relative distribution of saccade amplitude. Each participant's saccade amplitude falling within every 0.5° bin, within a range of 0–15°, was calculated. The graph displays the means among participants, with a representative value of each bin placed at its minimum (e.g., the value of a 10-10.5° bin is shown at 10° on the horizontal axis). (top left [A] and right [B]) distributions with a low-visibility window. (bottom left [C] and right [D]) distributions with a high-visibility window.

Supplementary Table 4. Estimated covariance parameter and fixed coefficients for the authorship questionnaire, comparing the self to the delay response.

| Covariance parameter | Estimate | Standard error | Z score | P value |
| --- | --- | --- | --- | --- |
| Participants | 1.347 | 0.627 | 2.147 | .032 |
| Fixed effects | Coefficient | Standard Error | T score | P value |
| Intercept | 1.827 | 0.496 | 3.686 | .001 |
| Delay | -0.018 | 0.002 | -8.913 | .000 |

Supplementary Table 5. Estimated covariance parameter and fixed coefficients for the authorship questionnaire, comparing the other to the delay response.

| Covariance parameter | Estimate | Standard error | Z score | P value |
| --- | --- | --- | --- | --- |
| Participants | 0.708 | 0.474 | 1.494 | .135 |
| Fixed effects | Coefficient | Standard Error | T score | P value |
| Intercept | -3.387 | 0.507 | -6.683 | .000 |
| Delay | 0.000 | 0.002 | 0.122 | .903 |

Supplementary Table 6. The results of the one-way multivariate ANOVA for the fitted slope values ($a$) and threshold delay values ($b$) within the delayed trials with the experiment type as a between-subjects factor.

|  | Pillai's Trace  Value | | df | F | | Error df | | P | | $\eta_{p}^{2}$ | | Noncent. Parameter | | Observed  Power |
| --- | --- | --- | --- | --- | --- | --- | --- | --- | --- | --- | --- | --- | --- | --- |
| Intercept | .912 | 4.000 | | 33.736 | 13.000 | | .000 | | .912 | | 134.944 | | 1.000 | |
| Experiment | .124 | 4.000 | | .459 | 13.000 | | .764 | | .124 | | 1.838 | | .125 | |

Supplementary Table 7. The results of the one-way ANOVA with repeated measures on mean authorship rating score.

|  | df | F | Error df | P | $\eta_{p}^{2}$ | Noncent. Parameter | Observed Power |
| --- | --- | --- | --- | --- | --- | --- | --- |
| Delay | 1.303 | 65.974 | 13.025 | .000 | .868 | 85.934 | 1.000 |

Supplementary Table 8. The results of the target × delay repeated-measures ANOVA on the response time

|  | df | F | Error df | P | $\eta_{p}^{2}$ | Noncent. Parameter | Observed Power |
| --- | --- | --- | --- | --- | --- | --- | --- |
| Delay | 1.734 | 33.814 | 17.341 | .000 | .772 | 58.635 | 1.000 |
| Target | 1.000 | 23.471 | 10.000 | .001 | .701 | 23.471 | .991 |
| Delay × Target | 2.393 | 2.358 | 23.931 | .108 | .191 | 5.643 | .468 |

Supplementary Table 9. The results of the one-way ANOVA with repeated measures on fixation counts per trial.

|  | df | F | Error df | P | $\eta_{p}^{2}$ | Noncent. Parameter | Observed Power |
| --- | --- | --- | --- | --- | --- | --- | --- |
| Delay | 1.751 | .774 | 17.512 | .460 | .072 | 1.356 | .154 |

Supplementary Table 10. The results of the two-way mixed ANOVA for the fixation count per second within the delayed trials. The delay variable was considered as a within-subjects factor, and experiment type (constant and jittery) as a between-subjects factor.

| Between-subject effect | df | F | P | $\eta_{p}^{2}$ | Noncent. Parameter | Observed Power |
| --- | --- | --- | --- | --- | --- | --- |
| Intercept | 1.000 | 1245.617 | .000 | .987 | 1245.617 | 1.000 |
| Experiment | 1.000 | .961 | .341 | .053 | .961 | .152 |
| Error (Experiment) | 17.000 |  |  |  |  |  |
| Within-subject effect | df | F | P | $\eta_{p}^{2}$ | Noncent. Parameter | Observed Power |
| Delay | 1.473 | 90.652 | .000 | .842 | 133.493 | 1.000 |
| Delay × Experiment | 1.473 | .416 | .603 | .024 | .613 | .103 |
| Error (Delay) | 25.034 |  |  |  |  |  |

Supplementary Figure 8. Comparison of the fitted parameter (a) on two categorical authorship responses (self and delay) between jitter and constant delays. The grey circle dots represent raw data of the fitted slope values (a) of ‘self’ responses from both the current experiment (N = 11) and the previous experiment (N = 8). The black squares indicate the average value at each experiment.

Supplementary Figure 9. Comparison of the fitted parameter (b) on two categorical authorship responses (self and delay) between jitter and constant delays. The grey circle dots represent raw data of the fitted threshold delay values (b) of ‘self’ responses from both the current experiment (N = 11) and the previous experiment (N = 8). The black squares indicate the average value at each experiment.
